# Self-grafting copper oxide nanoparticles show a strong enhancement of their anti-algal and anti-yeast action†

**DOI:** 10.1039/c9na00099b

**Published:** 2019-05-07

**Authors:** Ahmed F. Halbus, Tommy S. Horozov, Vesselin N. Paunov

**Affiliations:** Department of Chemistry and Biochemistry, University of Hull Hull HU67RX UK v.n.paunov@hull.ac.uk +44 (0)1482 465660; Department of Chemistry, College of Science, University of Babylon Hilla Iraq

## Abstract

We have developed and tested copper oxide nanoparticles (CuONPs) grafted with (3-glycidyloxypropyl)trimethoxysilane (GLYMO) and coupled with 4-hydroxyphenylboronic acid (4-HPBA), which provides a very strong boost of their action as anti-algal and anti-yeast agents. The boronic acid terminal groups on the surface of the CuONPs/GLYMO/4-HPBA can form reversible covalent bonds with the diol groups of glycoproteins and carbohydrates expressed on the cell surface where they bind and accumulate, which is not based on electrostatic adhesion. Results showed that, the impact of the 4-HPBA grafted CuONPs on microalgae (*C. reinhardtii*) and yeast (*S. cerevisiae*) is several hundred percent higher than that of bare CuONPs and CuONPs/GLYMO at the same particle concentration. SEM and TEM imaging revealed that 4-HPBA-functionalized CuONPs nanoparticles can accumulate more on the cell walls than non-functionalized CuONPs. We found a marked increase of the 4-HPBA functionalized CuONPs action on these microorganisms at shorter incubation times compared with the bare CuONPs at the same conditions. We also showed that the anti-algal action of CuONPs/GLYMO/4-HPBA can be controlled by the concentration of glucose in the media and that the effect is reversible as glucose competes with the diol residues on the algal cell walls for the HPBA groups on the CuONPs. Our experiments with human cell lines incubated with CuONPs/GLYMO/4-HPBA indicated a lack of measurable loss of cell viability at particle concentrations which are effective as anti-algal agents. CuONPs/GLYMO/4-HPBA can be used to drastically reduce the overall CuO concentration in anti-algal and anti-yeast formulations while strongly increasing their efficiency.

## Abstract 2

We report a strong amplification of the anti-algal and anti-yeast action of CuO nanoparticles surface-grafted with 4-hydroxyphenylboronic acid functional groups due to their covalent binding to carbohydrates on the cell membranes.

## Graphical abstract

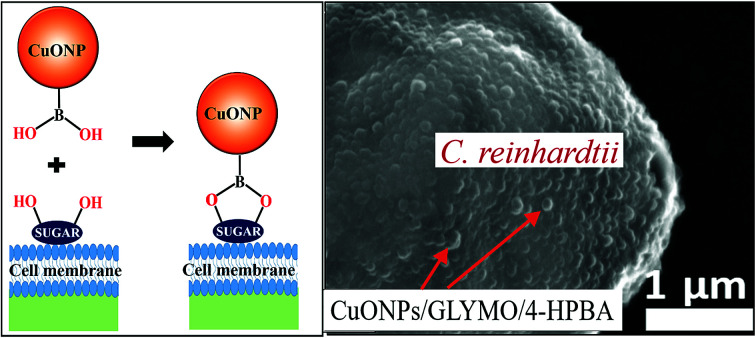

## Introduction

Over recent years there has been a substantial drive to find new approaches for developing novel antimicrobials as well as antialgal and antifungal agents.^1^ Colloidal particles of various materials have been recognized as promising and universal antimicrobials and antifungals as they do not rely on existing pathways of antimicrobial action and therefore should not be inhibited by antimicrobial resistances.^2^ Nanoparticles have been extensively used for an array of biological and medical applications, as contrast agents for medical imaging, labelling of cells, targeting of tumours and therapeutic drug delivery.^3,69–71^ This is due to their unique optical, photoactive, electronic, catalytic and thermal properties. These characteristics are variable for each role and can be often improved by possessing a specific particle morphology (sphere, cube, rod, *etc.*) and size.^4–6^ Often the nanoparticle shape and size can be easily controlled with a high degree of accuracy during their synthesis procedure.^7–16,69–71^ Metallic and metal oxide nanoparticles have been heavily researched in recent years for their nanotoxicity and potential antimicrobial capabilities due to their high surface area to volume ratios.^17–19,68^

CuONPs have been explored in various applications in biological research,^20^ as dopant for semiconductors, chemical sensors, supported heterogeneous nano-catalysts, as a coating material and in anti-cancer treatments.^21^ Lazary and co-workers have stated that CuONPs have been widely used in hospitals as anti-microbial agents due to their ability to kill more than 99.9% of Gram-negative and positive bacteria within 2 h of incubating if a suitable dose is used. It has been found that the use of CuO in this way has radically decreased the occurrence of hospital-acquired infections and the costs associated with health care. A non-intravenous approach to utilizing CuONPs in bed sheets is a very exciting innovation as the particles can decrease microbial attachment and therefore limit microbial infections within hospitals.^22^

Al-Awady *et al.* studied the antimicrobial effect of titania nanoparticles (TiO_2_NPs) of various particle sizes towards *C. reinhardtii* and *S. cerevisiae* upon illumination with UV and visible light for a range of nanoparticle concentrations and incubation times.^23^ They also confirmed that bare TiO_2_NPs affect the *C. reinhardtii* cells viability at much lower particle concentrations than for *S. cerevisiae*. The antimicrobial action of TiO_2_NPs increased slightly upon illumination with UV light compared with that in dark conditions due to the additional oxidative stress of the produced reactive oxygen species (ROS). They found that TiO_2_NPs have also affected *C. reinhardtii* upon illumination with visible light which indicates that they may also interfere with the microalgae's photosynthetic system leading to decreased chlorophyll content upon exposure to TiO_2_NPs.

Smaller TiO_2_NPs were found to have stronger antimicrobial effect, with anatase TiO_2_NPs generally being more effective than rutile TiO_2_NPs.^23^ The effect of nanoparticles (NPs) on different microorganisms depends on the particle size, morphology, synthesis method, the organism species, and as well as their surface chemistry. Aruoja *et al.* have studied the efficiency of three types of metal oxide nanoparticles (CuONPs, ZnONPs and TiO_2_NPs) in inhibiting the growth of microalgae *Pseudokirchneriella subcapitata*.^24^ Heinlaan and others have used the same three types of nanoparticles and found that CuONPs and ZnONPs have a toxic impact on *Thamnocephalus platyurus*, the bacteria *Vibrio fischeri* and crustaceans *D. magna*, while TiO_2_NPs were apparently non-toxic.^25^ Kasemets *et al.*^26^ examined the toxicity of CuONPs, TiO_2_NPs and ZnONPs on *S. cerevisiae* – a unicellular eukaryotic microorganism for 24 h of incubation. It was found that for *S. cerevisiae* both ZnONPs and bulk ZnO were of equivalent toxicity, while, CuONPs showed nearly 60-fold increase in cytotoxicity compared to the bulk CuO. It was discovered that both TiO_2_NPs and bulk TiO_2_ were non-toxic even at 20 000 μg L^−1^.

Limited research has so far been undertaken on the anti-yeast activity of copper, however, it is widely accepted that its usability against yeast is similar to that against bacterial species.^49,50^ The mechanism of ‘contact killing’ in *S. cerevisiae* and *C. albicans* cells has been investigated when in contact with Cu-based particles (C11000 99.9% Cu and C75200 62% Cu).^49^ By modifying Cu homeostasis, it was found that the elimination of *C. albicans* was 4–6 times faster, when compared to Cu ATPase export and *S. cerevisiae* deficient for Cu uptake transporters. Both scenarios involved the intracellular regulation of Cu rather than wild-type cells due to a large accumulation of Cu. This research group showed that the initial damages are localized on the cellular membranes, hence it action is similar to the ‘contact killing’ mechanism previously mentioned for bacteria. Characterization of the cell *via* mutation detection assays proved that there was an absence of DNA damage after treatment with Cu in this way. This did show extensive cytoplasmic membrane damage when the yeast was exposed to Cu surfaces. For the case of *C. albicans* strains, there were very high levels of the CRP1 P1-type ATPase copper transporter gene. By altering the intracellular uptake of Cu, there is a greater resistance against the Cu itself. An alternative resistance mechanism in place of the ‘contact killing’ scenario is suggested through the ALS1 and ALS3. This is a cluster of genes that encode the cell surface-associated glycoproteins, this could regulate the CRP1.^49,50^

Non-coated CuONPs have positive surface charge at neutral pH and can electrostatically adhere to the negatively charged cell walls. The average size of CuONPs is likewise essential for their potential anti-algal activity, as smaller nanoparticles have higher portability between biological compartments. However, the electrostatic interactions can be potentially modified and disrupted by the presence of another type of anionic species in the media, as, surfactants, polymers, proteins and others. This can result in the formation of carbohydrates and proteins corona which may change and even reverse the positive surface charge of the nanoparticles and render them ineffective as antialgal agents.^56–60^

**Fig. 1:**
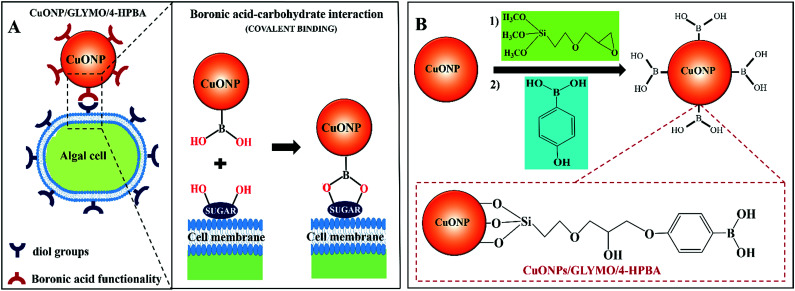
(A) Schematics showing the mechanism of self-grafting/covalent attachment of HPBA-functionalized CuONPs and the algal cell membrane. (B) The schematic of the synthesis method of CuONPs/GLYMO/4-HPBA by sequential grafting of GLYMO and 4-HPBA on CuONPs in an aqueous suspension.

In order to address this problem here we engineered CuONPs with a special coating containing terminal boronic acid surface groups. These were designed to provide a non-electrostatic mechanism for their attachment to the algae and yeast which was expected to enhance their accumulation on the cell walls even in the presence of anionic species in the media. We illustrate this design schematically in Fig. 1. Our idea is that the hydroxy phenyl boronic acid groups on the CuONPs will be able to covalently bind to various glycoproteins and carbohydrates that are abundant on the algal cell walls, thus forming boronic ester bonds with diols.^29,30^ Such boronic acid (BA) surface functionality has been used to prepare chemosensors for sugar groups^27^ and it is known that the BA makes them very effective for biomedical applications due to their low toxicity.^28,31^ Although this approach has been used for sensing, targeting and quantification of bacteria whose membranes contain various polysaccharides with diol groups,^32–37,68^ this is the first report where this functionality is used in the development of more effective anti-algal and anti-yeast nanoparticles.

Here we investigate the effect of (i) the bare CuONPs, CuONPs/GLYMO and CuONPs/GLYMO/4-HPBA particle concentration and (ii) the zeta potential and particle size on the viability of *C. reinhardtii* and *S. cerevisiae* at different exposure times under UV, visible light and in dark conditions. In this study we are interested in using the surface functionalized CuONPs as innovative anti-algal and anti-fungal agents. Since *C. reinhardtii* is a typical representative of the algae group and *S. cerevisiae* is a fungal microorganism, they are a good proxy for these assessments. Our results shed light on the possible mechanisms of their anti-algal and anti-yeast activity. The novelties of our approach is that CuONPs/GLYMO/4-HPBA target the cells by covalently binding to the glycoproteins and carbohydrates expressed on their membranes which does not rely on electrostatic interactions (Fig. 1A). Such self-grafting mechanism of attachment to cells is expected to amplify their antimicrobial action.

## Materials and methods

### Materials

We used copper(ii) chloride (99%, Sigma Aldrich) as a precursor in the synthesis CuONPs by direct precipitation with sodium hydroxide (99.6%, Fisher Scientific, UK). (3-Glycidyloxypropyl)trimethoxysilane (GLYMO) and 4-hydroxyphenylboronic acid (4-HPBA) were supplied from Sigma Aldrich. Fluorescein diacetate (FDA, 98%) for cell viability assays was purchased from Fluka, UK. *C. reinhardtii* cc-124 microalgae strain was kindly provided by Prof Flickinger's group at North Carolina State University, USA.

This microalgae culture was grown in Tris–Acetate–Phosphate (TAP) culture medium and incubated at 30 °C. The *C. reinhardtii* culture media consisted of TAP salts (NH_4_Cl; MgSO_4_·7H_2_O and CaCl_2_·2H_2_O), phosphate buffer solution (PBS) and Hutner's trace elements solution (EDTA disodium salt, ZnSO_4_·7H_2_O, H_3_BO_3_, MnCl_2_·4H_2_O, CoCl_2_·6H_2_O, CuSO_4_·5H_2_O, FeSO_4_·7H_2_O, (NH_4_)_6_Mo_7_O_24_·4H_2_O), all purchased from Sigma-Aldrich, UK. The microalgae batch was grown in the TAP media at pH 7 while being illuminated for 72 hours with a white luminescent lamp with a light intensity of 60 W m^−2^ under constant stirring with a magnetic stirrer.^61–63^ The stock cultures of *C. reinhardtii* were with a typical concentration of 4 × 10^5^ cells per mL determined by a cell counter (Nexcelom Cellometer Auto X4). *Saccharomyces cerevisiae* (Baker's yeast), was purchased from Sigma-Aldrich. It was cultured by hydrating 10 mg of lyophilized yeast cells in 10 mL of deionized water. Then 1 mL of this hydrated yeast cell suspension was added to 100 mL of autoclaved YPD culture media consisting of peptone (Sigma Aldrich, UK), d-glucose, (Fisher Scientific, UK), and yeast extract, (Oxoid ltd, UK), then incubated at 30 °C for 24–48 hours.^64^ Deionized water from a Milli-Q reagent water system (Millipore, UK), was used in all experiments.

### Methods

#### Synthesis and characterization of CuONPs

A sample of 3.0 g of copper(ii) chloride (CuCl_2_) was dissolved in 160 mL of ethanol and 1.8 g of sodium hydroxide (NaOH) was dissolved in 50 mL ethanol, separately. The NaOH solution was added dropwise to the CuCl_2_ solution at constant stirring at room temperature. The mixed solution colour changed from green to greenish blue and finally yielded a black precipitate of copper(ii) hydroxide (Cu(OH)_2_) – see Fig. S1† for details of the synthesis scheme. The precipitate was then settled using a centrifuge, washed with ethanol and deionized water and dried at 60 °C in an electric furnace. In order to produce CuONPs, the sample of dried copper hydroxide was annealed at several different temperatures of 100 °C, 200 °C, 300 °C, 400 °C, 500 °C and 600 °C, respectively and finally grinded to a powder.^38^ The CuONPs were produced by dispersing the obtained CuO powder in deionized water at pH 6 *via* sonication (Branson sonicator 420) for 10 min at 40% of the maximum power. The characterization of the CuONPs size and zeta potential distribution in aqueous solutions was done with a Zetasizer Nano ZL (Malvern, UK).

#### Grafting of CuONPs with GLYMO and 4-HPBA

0.1 g of CuO was dispersed into 100 mL of deionized water of pH 6–6.5 by sonication followed by addition of 0.1 g GLYMO. The reaction mixture was stirred for 24 h, then the excess GLYMO was removed by centrifugation and washing three times with water.^39^ The GLYMO-grafted CuONPs were then re-dispersed in 100 mL of deionized water and then mixed drop-wise with 0.1 g of 4-HPBA dissolved in 100 mL of ethanol solution, shaken for 2 hours and triple washed with ethanol at 10 000 rpm for 30 min.

The CuONPs/GLYMO/4-HPBA were finally re-dispersed in 100 mL of deionized water^40^ using a digital sonicator Branson 420 at 40% of the maximum power for 15 min (2 s ON–2 s OFF pulse time). The particle size and the zeta potential of the GLYMO/HPBA grafted CuONPs was examined by dynamic light scattering (DLS) using the Malvern Zetasizer Nano ZL. All examinations were done at 25 °C and the results reported are an average of 3 readings. pH of the solutions was varied from 3 to 12 (using 0.1 M HCl and 0.1 M NaOH) and adding two drops of 0.01 M NaCl into each sample (10 mL).

#### Anti-algal and anti-yeast activity of HPBA-grafted CuONPs

UV lamp and white light lamp were used to illuminate the samples in this study. UV light illumination was carried out with the radiation source type 11868010, UVP™ Fraud Detection Lamp, 6 W (Fisher Scientific, UK). Aqueous suspensions of the nanoparticles containing cells, under magnetic stirring, were irradiated with 365 nm UV light at an irradiation intensity of 161 ± 5 Lux. Visible light illumination was carried out using a Maxibright T5 lamp (Germany) in all our studies. The distance from the source (both UV and visible lamp) was 14 cm. 50 mL of *C. reinhardtii* cells were washed three times from the culture media and re-dispersed in 30 mL deionized water. 5 mL aliquots of the washed *C. reinhardtii* cells suspension were incubated with a series of 5 mL aliquots of the aqueous dispersions of CuONPs (bare, GLYMO- or HPBA/GLYMO-grafted) at different particle concentrations. After that, these samples were split into three equal parts which were illuminated for various exposure times under visible light or UV light, or kept in dark conditions, respectively. Likewise, a control sample of the cells was treated at the similar conditions without exposure to CuONPs. After that, 1 mL of the *C. reinhardtii* suspension was taken from each tested sample, washed with deionized water to remove the excess of CuONPs by centrifugation at 3500 rpm for 4 minutes and re-suspended in 1 mL of deionized water. Two drops of 1 mM FDA solution in acetone was added to each sample and mixed together for 15 min. After that, these samples were washed three times with deionized water by centrifugation at 3500 rpm for 4 min to remove the excess of FDA. Finally, the cell viability was tested by using automatic cell counter. The same methodology was used to test the impact of CuONPs functionalized with GLYMO and 4-HPBA on the cell viability of *C. reinhardtii*, which were incubated with different concentrations for various exposure times.

The effect of CuONPs on *S. cerevisiae* was also examined, by the following procedure. A 30 mL aliquot of the *S. cerevisiae* culture was washed three times with deionized water *via* centrifugation and after that re-dispersed in 30 mL deionized water. Then, 5 mL of *S. cerevisiae* dispersion were mixed with 5 mL of the CuONPs aqueous suspension at various total particle concentrations. After that, the tested suspensions were exposed separately for various incubation times under UV light or visible light, or kept in dark condition. 1 mL of each sample was taken from each tested sample with CuONPs and the cells were washed with deionized water *via* centrifugation at 3500 rpm for 4 minutes to remove the excess of CuONPs. The *S. cerevisiae* cells were re-suspended in 1 mL of deionized water and then 2 drops of FDA solution were added to each sample and mixed together for 15 min with a magnetic stirrer. After that, the samples were washed three times with deionized water *via* centrifugation at 3500 rpm for 4 min. Finally, the viable percentage of cells was examined by using 20 μL of the cell suspension with an automatic cell counter Cellometer Auto X4 fitted with a fluorescein filter set.

#### Anti-algal and anti-yeast activity of free GLYMO and 4-HPBA

Fig. S13 and S14† shows the cytotoxicity assay of the free GLYMO and 4-HPBA on *C. reinhardtii* and *S. cerevisiae* for up to 2 h for *C. reinhardtii* and 6 h for *S. cerevisiae* of exposure. Both runs were done at the varying overall GLYMO and 4-HPBA concentration and different incubation times. One can see a very small effect on the presence of free GLYMO on the *C. reinhardtii* viability over a period of up to 2 hours (Fig. S13A†). Hence the free GLYMO and 4-HPBA does not measurably impact the cell viability up to 25 μg mL^−1^. Note that in our CuONPs/GLYMO/HPBA nanoparticles there is not any free HPBA and free GLYMO as the particles have undergo multiple washing/centrifugation cycles after their surface functionalization. However, at these concentrations of the HPBA-grafted on CuONPs, the effect of the CuONPs on *C. reinhardtii* and *S. cerevisiae* is very significant – see Fig. 4 and 8, respectively. Therefore, one may conclude that the HPBA-grafted CuONPs shows excellent anti-algal and anti-yeast activity which is not related to the presence of free HPBA.

#### SEM and TEM sample preparation protocol for *S. cerevisiae* and *C. reinhardtii* after exposure to CuONPs

After incubation with the CuONPs, the *S. cerevisiae* and *C. reinhardtii* were fixed for 2 h with 2 wt% glutaraldehyde at room temperature in 0.1 M cacodylate buffer at pH 7.2. These samples were post-fixed in 1% osmium tetroxide for 1 h, and after that samples were dehydrated in a range of ethanol solutions with concentrations up to 100%. Finally, samples were dried at critical point using liquid carbon dioxide.^41,42^ The microorganisms before and after treatment with the CuONPs (bare and HPBA-grafted) were imaged using a SEM. After incubation with CuONPs, the cells were studied with TEM *via* the following procedure. These microorganisms examined were washed with deionized water by centrifugation to remove the CuONPs suspension at 500 × *g* and fixed in 2 wt% glutaraldehyde for one hour at room temperature followed by treatment with 1 wt% osmium tetra-oxide for one hour. Then, these microorganisms were treated with 2.5 wt% uranyl acetate for one hour, then washed with ethanol solutions of increasing concentration. After standard dehydration, the *S. cerevisiae* and *C. reinhardtii* samples were embedded in fresh epoxy/Araldite at 60 °C for 2 days, left for 2 days at room temperature and then sectioned using an ultra-microtome. After that, the microorganisms before and after treatment with inorganic nanoparticles were imaged by TEM.

#### Cytotoxicity assay of bare- and HPBA-grafted CuONPs on human embryonic kidney cells

HEK 293 cell line culture (sourced from Public Health England) was grown in high-glucose DMEM media supplemented with 10% Fetal Bovine Serum (FBS, Labtech, UK) and 1% antibiotics (Penicillin Streptomycin, Lonza, UK) inside an incubator (37 °C, 5% CO_2_). After reaching 70% confluence, HEK 293 cells were carefully washed with PBS for 10 seconds then incubated with 0.25% Trypsin–EDTA (1×, Lonza, UK) to detach the cells from their support after 5 minutes. The Trypsin–EDTA action was neutralized by adding complete DMEM medium before a centrifugation at 400 × *g* for 4 minutes. A 25 mL aliquot of the HEK 293 cells culture (∼70 000 cells per mL) was washed three times from the culture media by centrifugation and re-dispersed in 25 mL PBS. Then, 2.5 mL aliquots of this HEK cells suspension were incubated with a series of 2.5 mL aliquots of aqueous dispersions of bare and HPBA-grafted CuONPs at different concentrations. Likewise, a control sample of the HEK cells was treated at the similar conditions without exposure to any nanoparticles. After that, 1 mL of the solution HEK was taken from each sample treated with nanoparticles, washed with PBS to remove the excess of nanoparticles by centrifugation at 400 × *g* for 4 min. The HEK cells were re-suspended in 1 mL of PBS, then two drops of FDA solution in acetone were added to each sample, mixed together for 15 min and washed three times with PBS by centrifugation at 400 × *g* for 4 min. A microplate reader was utilized to assay the HEK cell viability.

## Results and discussion

### Characterization of GLYMO- and HPBA-grafted CuONPs

The crystalline nature of CuONPs was studied by X-ray diffraction (XRD). Fig. 3D shows XRD pattern of CuONPs obtained by direct precipitation method using a copper chloride solution after annealing at 100 °C (see Fig. S5 and S6, ESI†). The diffraction peaks correspond to the hexagonal structure of CuO according to Joint Committee on Powder Diffraction Standards (JCPDS no. 01-077-7716). This indicates that are no apparent impurities, suggesting that CuO of high purity has been prepared. The average crystallite size of the CuO can be calculated using the Scherrer equation. Fig. 3E shows the EDX analysis data obtained at 10 keV from CuONPs annealed at 100 °C. The results reveal the presence of copper (Cu) and oxygen (O) without other detectable elemental impurities in the EDX spectra. Note that there is a small peak of carbon due to the carbon coating of the sample prepared for SEM imaging. The elemental analysis confirmed that the synthesized sample was CuO, which is in a good agreement with the results of XRD and the literature.^43,44^ The average crystallite size of the CuONPs derived from XRD measurements was about 13 nm from XRD data while their average hydrodynamic diameter measured by DLS was about 93 nm with a surface charge around +37 mV as presented in Fig. S2 and S4 (ESI†). This indicates that the actual CuONPs in aqueous suspension are aggregates of many individual crystallites with overall irregular shape.

**Fig. 2:**
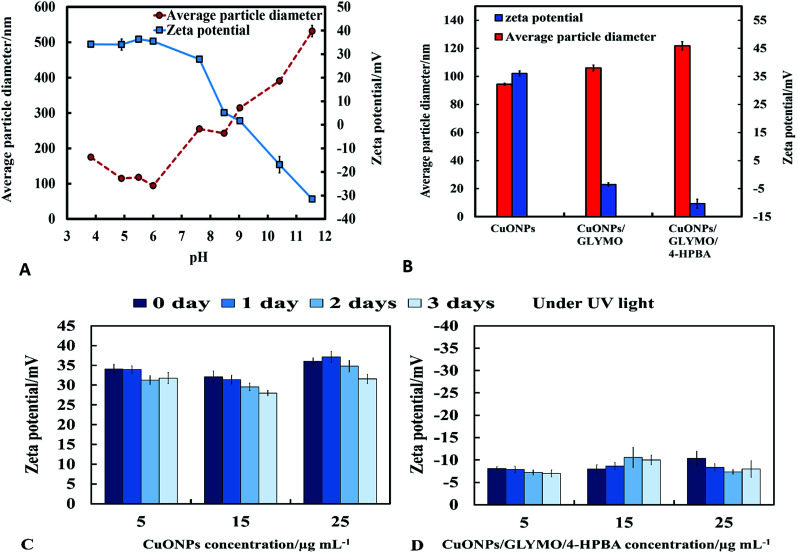
(A) Zeta potential and particle size of CuONPs dispersed in aqueous solution *versus* pH. (B) Zeta potential and hydrodynamic diameter of the surface modified CuONPs with GLYMO and 4-HPBA, measured at pH 6 at room temperature (error bar represents standard deviation derived from three DLS size distribution measurements). The zeta potential of (C) bare CuONPs and (D) CuONPs grafted with GLYMO and 4-HPBA at different concentrations (5, 15 and 25 μg mL^−1^) after exposure to UV light for 0 day, 1 day, 2 days and 3 days.

The mean hydrodynamic diameter and zeta potential of these CuONPs in deionized water was measured by dynamic light scattering (DLS). The isoelectric point of the CuONPs determines at what pH they are expected to be effective at killing the targeted microalgae and yeast. It can be seen from Fig. 2A that the point of zero zeta potential of the bare CuONPs is at pH 9 where the particle suspension is least stable. When the CuONPs are in an aqueous solution near the isoelectric point, they would partially aggregate due the lack of electrostatic repulsion between them. Furthermore, due to the aggregation, the particle surface morphology could affected their activity against the studied microorganisms. In order to avoid ambiguity in the interpretation of these results, we chose to carry out our tests away from the NPs isoelectric point. We used solutions with pH between 5 and 6 (typical for deionized water) to ensure that the NPs would not aggregate and maintain their average size around 100 nm. The same batch of bare CuONPs dispersions was subsequently used to prepare aqueous suspensions of CuONPs with GLYMO or HPBA grafted on their surface. Bare CuONPs dispersed in water had the smallest hydrodynamic diameter (94 ± 3 nm), while the size of surface-modified CuONPs varied between 106 ± 6 nm (CuONPs/GLYMO) and 121 ± 4 nm (CuONPs/GLYMO/4-HPBA) – see Fig. 2B. The zeta potential of the bare CuONPs was positive at pH 6 while both types of surface-modified CuONPs had negative zeta potentials, ranging from around −3 mV (CuONPs/GLYMO) to −10 mV (CuONPs/GLYMO/4-HPBA) (see Fig. 2B). Since the CuONPs are photoactive, there was a concern that their GLYMO/HPBA surface functionality can potentially be affected by oxidation under the action of UV light. To check the stability of this coating against oxidation, we measured periodically the zeta potential of the CuONPs/GLYMO/4-HPBA over the course of 3 days while the samples were exposed to UV light (365 nm, 161 ± 5 Lux). The results presented in Fig. 2C and D indicate that the zeta potential of the GLYMO/4-HPBA functionalized CuONPs does not change, *i.e.*, the coating is not prone to oxidation at these conditions, and hence the particles preserve their functionality as well as their anti-algal and anti-yeast action.

**Fig. 3:**
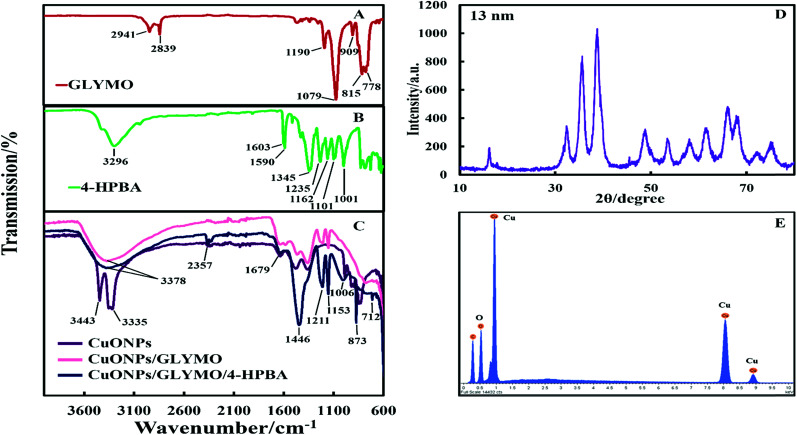
The FTIR spectra of (A) pure GLYMO, (B) pure 4-HPBA and (C) the bare CuONPs, GLYMO-grafted CuONPs and CuONPs grafted with GLYMO and 4-HPBA. (D) XRD pattern of CuONPs annealed at 100 °C. The largest peak in the XRD results was used to measure the crystallite size. (E) EDX analysis of CuONPs.

The hydroxyl groups on the surface of the CuONPs in aqueous solution are the binding sites for the reaction with alkoxyl groups of silane compounds. The efficiency of silane in grafting GLYMO and 4-HPBA on CuONPs was determined by FTIR. Fig. 3A–C shows normalized FTIR spectra of unmodified CuONPs (see Fig. S3, ESI†) and CuONPs surface modified with GLYMO and 4-HPBA. In the spectra of all CuONPs the broad band between 400 and 800 cm^−1^ correspond to Cu–O–Cu. GLYMO contains two functional groups, epoxy- and methoxysilyl, which can both hydrolyze and condensate. The epoxy-band in FTIR spectra is preserved, while the intensity of Si–O–Me band is decreased. The two bands of OH groups appear at ∼3300 and ∼1640 cm^−1^ because of hydrolysis of the Si–O–Me groups. Also, a peak at 1050 cm^−1^ appears, which can be assigned to the formation of Si–O–Si bonds. Compared with the spectrum of the bare CuONPs, the FTIR spectrum of GLYMO-grafted CuONPs sample shows some new characteristic absorption peaks. Fig. 3C (CuONPs/GLYMO) shows peak at ∼1200 cm^−1^ which refers to Si–O–Me groups.^45^ We also tried to characterize the surface structure of the CuONPs/GLYMO/4-HPBA. In the FTIR spectrum of the CuONPs/GLYMO/4-HPBA, the peak at about 3300 cm^−1^ could be attributed to the stretching vibration of the O–H groups. The peaks at ∼2500 cm^−1^ were assigned to the stretching and bending vibrations of the C–H groups. The bending of the aromatic C=C groups could be also observed at (1490–1650 cm^−1^). The sharp peaks at around 1343 cm^−1^ and 1090 cm^−1^ could be assigned to the stretching vibrations B–O and C–B groups (Fig. 3C).^46,47^

### Anti-algal activity of GLYMO- and HPBA-grafted CuONPs

**Fig. 4:**
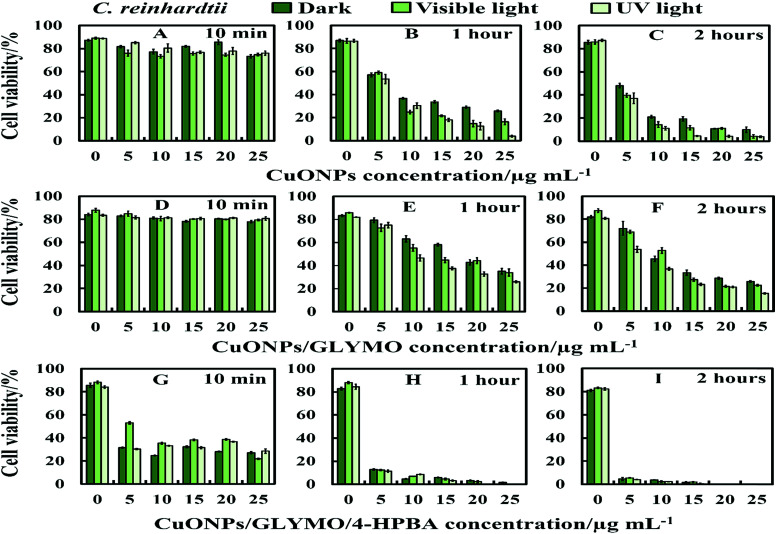
The antialgal activity of bare, GLYMO or 4-HPBA–GLYMO-functionalized CuONPs at various concentrations (0, 5, 10, 15, 20 and 25 μg mL^−1^) on *C. reinhardtii*. The *C. reinhardtii* was incubated with the nanoparticles at 10 min, 1 hour and 2 hours of exposure times in dark conditions, under visible and UV light. Statistical analysis of these data is enclosed in Table S1 (ESI†).

**Fig. 5:**
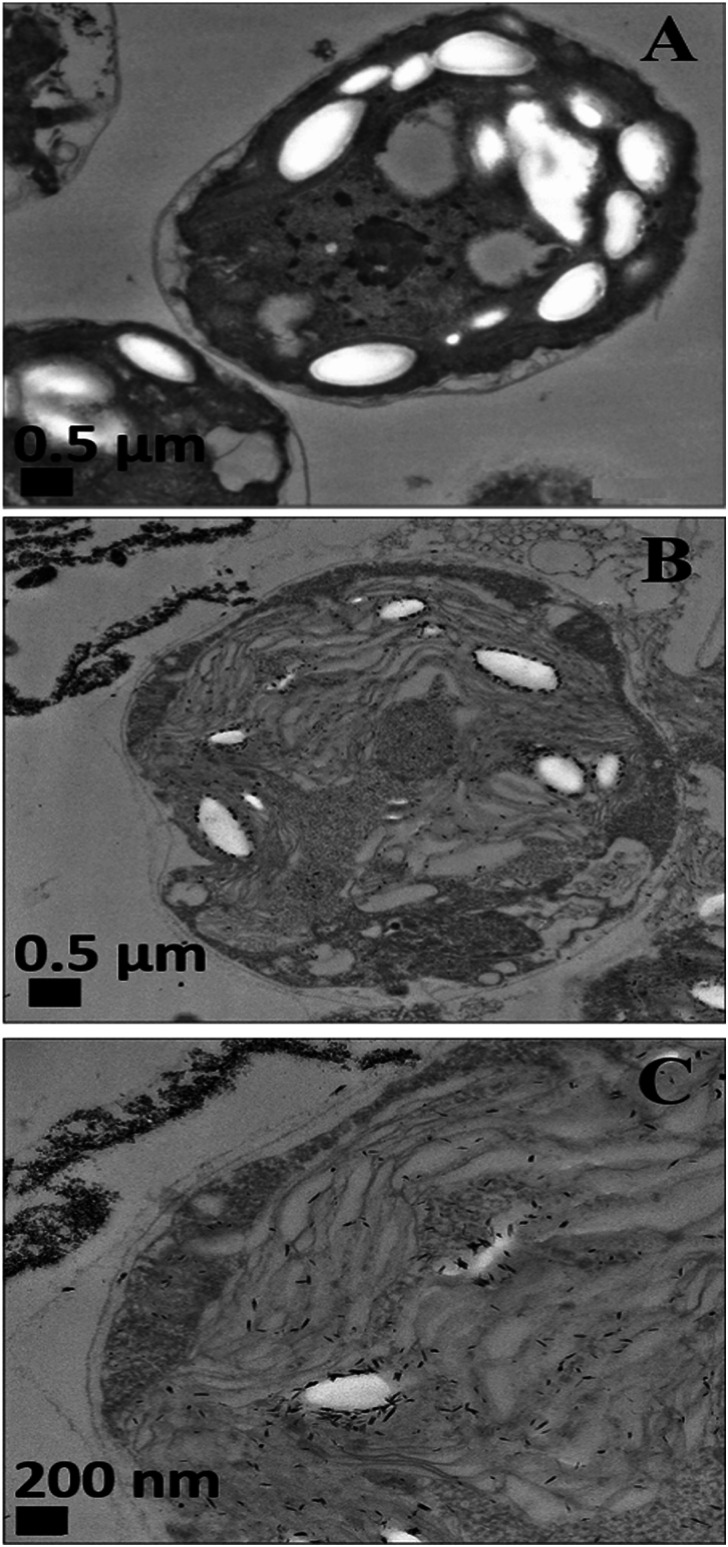
TEM images of *C. reinhardtii* after being exposed for 2 hours to CuONPs: (A) control of *C. reinhardtii* before treatment with CuONPs (B and C) *C. reinhardtii* after treatment with 25 μg mL^−1^ of CuONPs at different magnifications.

**Fig. 6:**
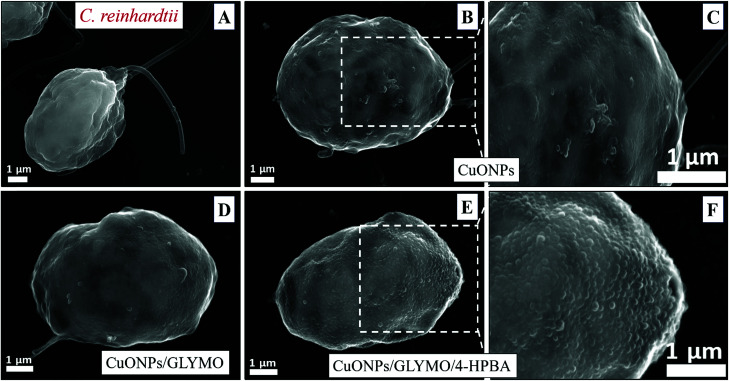
SEM images of *C. reinhardtii* after being exposed for 2 hours to nanoparticles: (A) a control of *C. reinhardtii* before treatment with CuONPs, (B and C) *C. reinhardtii* after treatment with 25 μg mL^−1^ of CuONPs, (D) *C. reinhardtii* after treatment with 25 μg mL^−1^ of CuONPs/GLYMO, (E and F) *C. reinhardtii* after treatment with 25 μg mL^−1^ of CuONPs/GLYMO/4-HPBA.

We conducted tests with *C. reinhardtii* and the HPBA-grafted CuONPs after removing the cells from their culture media. Fig. 4 compares the effect of bare CuONPs and surface-grafted CuONPs with GLYMO and 4-HPBA at different particle concentrations on the *C. reinhardtii* viability. During the first 10 min of exposure, the cells were not affected by both the bare CuONPs and CuONPs/GLYMO up to a concentration of 25 μg mL^−1^ (see Fig. 4A and D). However, the CuONPs/GLYMO/4-HPBA nanoparticles showed a significant impact on the algal cell viability even at this short exposure time (Fig. 4G). In this case, the algal cell viability decreased more than 5-fold upon exposure from 5 μg mL^−1^ to 25 μg mL^−1^ CuONPs/GLYMO/HPBA compared to the bare CuONPs. This can be attributed to several factors. First, *C. reinhardtii* cell walls consist of polysaccharides, glycoproteins, and cellulose, which provide multiple binding sites for the cationic CuONPs *via* nonspecific electrostatic interactions with the anionic surface of the cells. It is widely discussed in the literature that CuONPs have been appeared to create ROS under UV light due to their pronounced photocatalytic properties in aqueous solution.^2^ Since the pore sizes in the plant cell walls are commonly in the range 5–20 nm,^48^ the CuONPs (93 nm in diameter) adsorbed onto the algal surfaces-as confirmed by the TEM (Fig. 5B and C) and SEM imaging (Fig. 6B and C) are unlikely to permeate through the *C. reinhardtii* cell walls.

Nevertheless, the accumulation of nanoparticles could impact mechanically the *C. reinhardtii* cell membranes where CuONPs are clustered as a rough aggregate which could cause braking of the membrane and discharge of the *C. reinhardtii* cell content to the extracellular space as shown in the EDX data (Fig. S7, ESI†). Moreover, the accumulation of densely packed CuONPs on the *C. reinhardtii* cell membrane could also block nutrient uptake, thus altering the photosynthetic efficiency of algae as evidenced in the previous studies.^48^ In extreme cases, this process can distort algal cell walls as implied by the severely wrinkled and deformed *C. reinhardtii* cell shown in TEM (Fig. 5B and C) and SEM images (Fig. 6B, C, E and F).

Various mechanisms have been discussed in the literature about how CuONPs kill algal cells and their antibacterial action might be a combination of all of them. One mechanism is based on the photoactive nature of these nanoparticles which in the presence of oxygen from air and visible or UV light, form ROS which are free radicals and lead to peroxidation of lipids from the bacterial cell membrane.^2,23,65,66^ The cell wall of the microalgae is negatively charged while the non-functionalized (bare) CuONPs are positively charged in water (below pH 9). Therefore, the bare CuONPs were able to electrostatically adhere on the bacterial cell surface which led to damage of their cell membrane. When the bare CuONPs attach to the cell, the ROS created locally under UV light can interact directly with the cell membrane and organelles which can amplify the cell damage. The ROS generation can also start a chain of free radical reactions inside the algae. Lipid membrane peroxidation is a type of oxidative stress for the algal cells, which leads to their loss of viability. On the other hand, Fig. 4 shows that the anti-algal activity of 25 μg mL^−1^ CuONPs under UV light for 1 hour is only slightly higher than that under dark conditions. This suggests that the ROS generation under UV light has only a little effect on the anti-algal action of CuONPs.

**Fig. 7:**
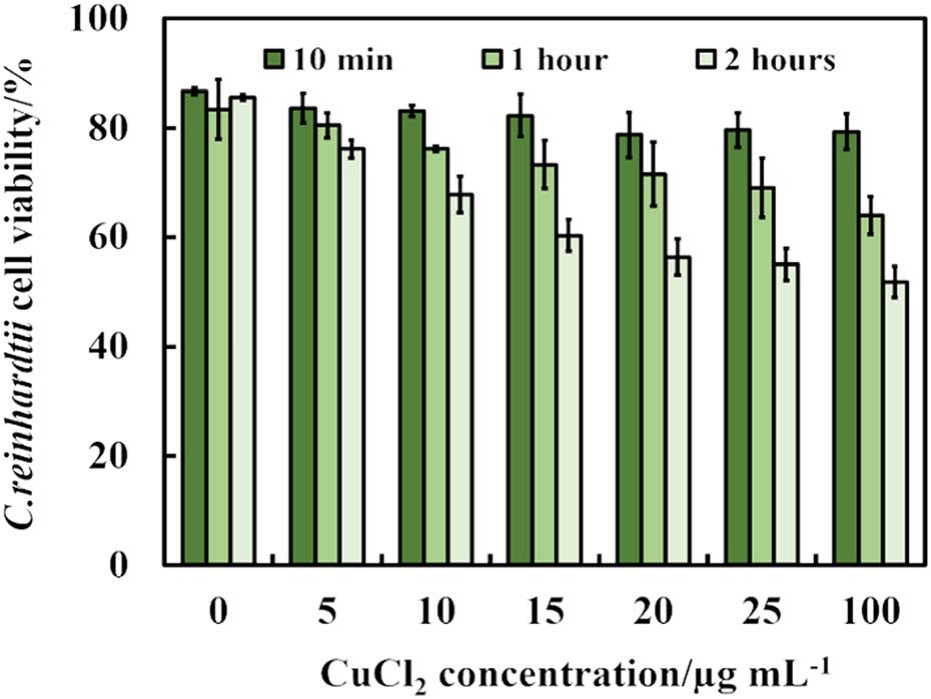
The *C. reinhardtii* cell viability after incubation with CuCl_2_ as a function of CuCl_2_ concentration for up to 2 hours.

Another possible antimicrobial mechanism is the release of free Cu^2+^ ions from the CuONPs which may interfere with the cell membrane proteins. However, the concentration of free Cu^2+^ ions in the aqueous solution around the CuONPs is negligible due to its very low solubility at pH 5–6. In order to investigate the effect of the presence of Cu^2+^ ions emitted from the CuONPs in aqueous solution on the viability of the *C. reinhardtii* microalgae, we tested a range of CuCl_2_ concentrations 0–100 μg mL^−1^. One can see from the results presented in Fig. 7 that the effect of the Cu^2+^ ions on the microalgae viability is ∼2 times smaller than the CuONPs/GLYMO/HPBA. The CuO solubility varies with pH but in deionized water at pH 6–6.5 it is approximately 2.5–3 × 10^−5^ M.^67^ Thus the presence of Cu^2+^ is not sufficient to explain the anti-algal effect of CuONPs, which increases with their concentration, while the CuO solubility is constant at fixed pH and temperature. Our current understanding is that the strong anti-algal action can be explained by the attraction between the cationic bare CuONPs with the anionic algal cell membrane. As the CuONPs are nano-aggregates with a rough surface, a likely explanation of the strong anti-algal effect is that their adhesion to the cell membrane causes its rupture and this is the main contributing factor to the cell death. This indicates that the covalent binding is the main reason for the buildup of CuONPs/GLYMO/4-HPBA on the algal surface, as confirmed by the TEM and SEM images in Fig. 5 and 6.

### Anti-yeast activity of GLYMO- and HPBA-grafted CuONPs

**Fig. 8:**
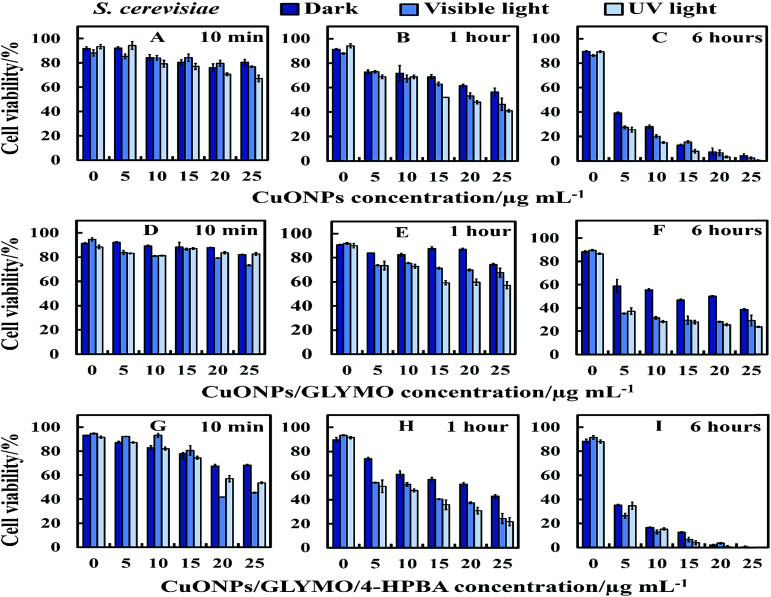
Representative the cell viability of *S. cerevisiae* cells upon incubation of bare and HPBA-surface grafted CuONPs of different particle concentrations (0, 5, 10, 15, 20 and 25 μg mL^−1^) in dark, visible and UV light conditions. The *S. cerevisiae* cells were incubated with: (A–C) bare CuONPs; (D–F) CuONPs/GLYMO and (G–I) CuONPs/GLYMO/4-HPBA at 10 min, 1 hour and 6 hours exposure times. Data are means ± SD of three independent replicates.

There are varying levels of anti-yeast activity for non-functionalized CuONPs and their functionalized versions, CuONPs/GLYMO, and CuONPs/GLYMO/4-HPBA. To investigate this effect on *S. cerevisiae*, we incubated yeast cells with bare CuONPs, GLYMO- and HPBA-surface grafted CuONPs at varying concentrations (0–25 μg mL^−1^). The results for the *S. cerevisiae* cell viability are presented in Fig. 8A–I and indicate lowering of the viability upon increasing the CuONPs concentration. For this study, the *S. cerevisiae* presented a level of resistance to CuONPs/GLYMO, whereas both bare CuONPs and CuONPs/GLYMO/4-HPBA showed a much higher activity when introduced to *S. cerevisiae*. In particular, the CuONPs/GLYMO/4-HPBA proved to be far more effective when the concentration was between 20 and 25 μg mL^−1^ (Fig. 8I). Many different studies on cells have also demonstrated that copper exposure rapidly prompts membrane alterations before DNA degradation.^53,54^ All these investigations concluded that the antimicrobial effect of copper was related to its ability to discharge copper ions and their damaging impact on the cell membrane. Other mechanisms for the antimicrobial effect of CuONPs have also been discussed which involve adhesion of CuONPs to cells by electrostatic interactions, similar to that in the algal studies.

CuONPs/GLYMO/4-HPBA can form reversible boronic ester interactions with *cis*-diol-containing carbohydrate and glycoproteins molecules which are abundant on the yeast cell wall.^55^

**Fig. 9:**
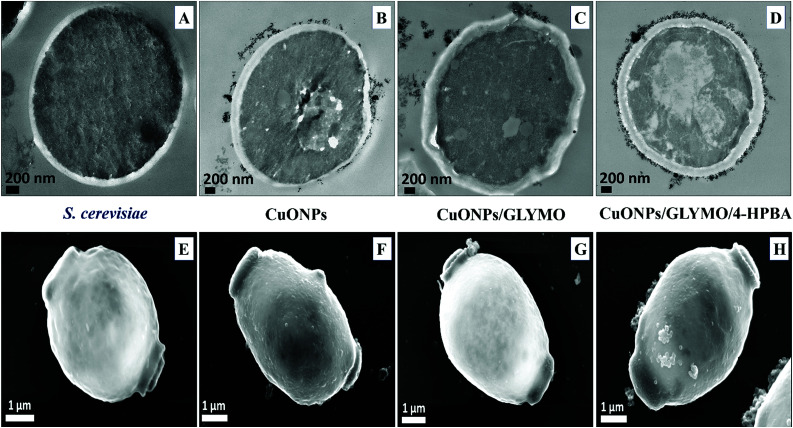
TEM and SEM image of *S. cerevisiae* cells with (A) and (E) being the control (untreated) sample, (B) and (F) samples of *S. cerevisiae* after incubation in a suspension of 25 μg mL^−1^ CuONPs for 6 h, (C) and (G) after incubation with 25 μg mL^−1^ CuONPs/GLYMO and (D) and (H) after incubation with 25 μg mL^−1^ CuONPs/GLYMO/4-HPBA.

Fig. 9 shows the TEM and SEM image of *S. cerevisiae* cells incubated with bare CuONPs as well as ones surface-grafted with GLYMO and 4-HPBA. Sectioned samples of the *S. cerevisiae* exposed to CuONPs were analyzed with EDX (Fig. S8, ESI†) and revealed that the CuONPs accumulate predominantly on the outer side of the cell membrane. TEM images (Fig. 9D) and SEM images (Fig. 9H) demonstrated an extracellular accumulation of CuONPs/GLYMO/4-HPBA which leads to cell death due to membrane damage. There are many studies suggesting that ‘contact killing’ is started by the dissolved Cu^2+^ ions discharged from the copper surfaces by the culture medium and causing cell damage by interacting with enzymes and DNA.^51,52^ However, as discussed earlier, the concentration of Cu^2+^ supported by CuONPs is apparently too low to produce this effect. Comparison of the anti-yeast activity of CuONPs/GLYMO and of CuONPs/GLYMO/HPBA indicate that the difference can be attributed to their cell binding ability rather than the Cu^2+^ ions, as they both emit the same concentration of Cu^2+^, determined by the CuO solubility product.

An additional confirmation for the mechanism of attachment of the CuONPs/GLYMO/4-HPBA to both algal and yeast cells is presented in Fig. S9–S12,† where we compared the zeta potential of these cells after being treated with bare CuONPs and CuONPs/GLYMO/4-HPBA at different particle concentrations. The zeta potential of both types of cells treated with bare CuONPs, which are cationic at neutral pH, is reduced by absolute value (Fig. S9 and S11, ESI†) due to the partial deposition of the cationic CuONPs on the negatively charged outer cells wall. However, the incubation of both types of cells with CuONPs/GLYMO/4-HPBA does not incur measurable change in their zeta-potential despite their adsorption on the cells wall (Fig. S10 and S12†). This is an additional confirmation that the attachment of the CuONPs/GLYMO/4-HPBA to both algae and yeast cells is not based on electrostatic attraction and the HPBA-grafted CuONPs bind to the cells despite their negative surface charge. Apparently, the negatively charged GLYMO-grafted CuONPs do not bind the cells although their surface charge is very similar to that of the CuONPs/GLYMO/4-HPBA. This indicates that the covalent binding is the main reason for the build-up of CuONPs/GLYMO/4-HPBA on the cells surface, as confirmed by the TEM and SEM images in Fig. 5, 6 and 9.

### Anti-algal and anti-fungal activity of HPBA-grafted CuONPs in the presence of glucose

**Fig. 10:**
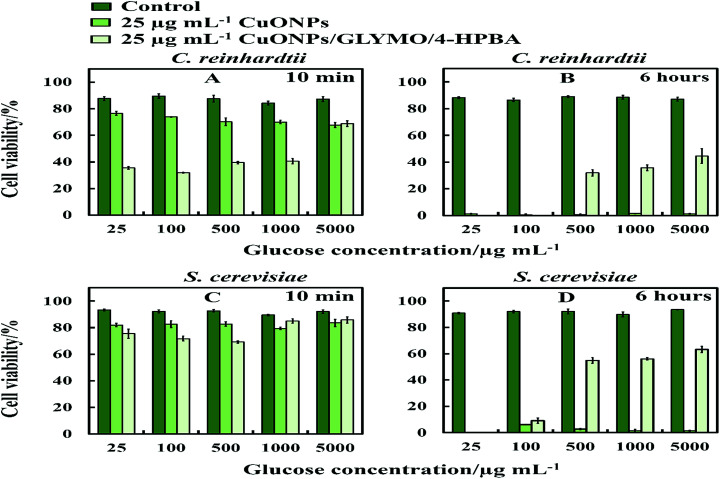
The cell viability after incubation as a function of nanoparticle concentration for 6 h at various glucose concentrations. (A and B) *C. reinhardtii* and (C and D) *S. cerevisiae* cells.

The anti-algal and antifungal activity of GLYMO- and HPBA-grafted CuONPs were studied towards *C. reinhardtii* and *S. cerevisiae* in the presence of different concentrations of glucose at 25 μg mL^−1^ nanoparticle concentration (Fig. 10). The glucose was added to the cell suspension before the addition of the bare CuONPs and CuONPs/GLYMO/4-HPBA suspension under dark conditions. We discovered that the cells apparently loss their viability in the presence of bare CuONPs after 6 hours not only upon exposure to lower concentrations of glucose, but also upon incubation with higher concentrations of glucose. We also found that the cell viability in the presence of CuONPs/GLYMO/4-HPBA nanoparticles increases with an increase of the glucose concentration. A possible mechanism for this could be that the hydroxyl groups of the 4-hydroxyphenylboronic acid on the surface of CuONPs/GLYMO/4-HPBA nanoparticles interact with the glucose, thus reducing the interaction between 4-HPBA-groups and the carbohydrates on the algal and yeast cell membranes.

### Toxicity of bare- and HPBA-grafted CuONPs on human cells

**Fig. 11:**
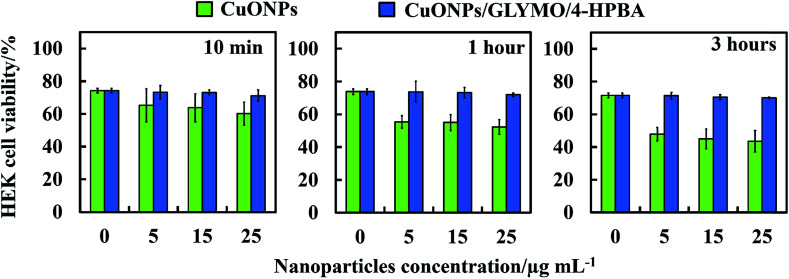
Comparison of the cell viability of human embryonic kidney cells (HEK 293 cell line) upon incubation as a function of nanoparticle concentration for up to 3 h at with bare CuONPs and CuONPs/GLYMO/4-HPBA under dark conditions.

We did some preliminary studies on the cytotoxicity of the HPBA-grafted CuONPs on representative samples of human cells. Fig. 11 shows the results on cytotoxicity assay of CuONPs and CuONPs/GLYMO/HPBA on HEK 293 cells (human embryonic kidney cell line) for up to several hours of exposure. The results confirm that CuONPs/GLYMO/HPBA had no measurable toxicity on these cells while with bare CuONPs some low level of toxicity was measured compared with the control sample (no CuONPs) for the duration of their exposure. These results were obtained with particle concentrations where they show very strong anti-algal and anti-yeast effect, respectively. This result is reassuring that such functionalized CoNPs particles can potentially find applications in anti-algal formulation at much lower concentration without potentially harmful effect to the environment and human health.

## Conclusions

In conclusion, we demonstrated that by surface grafting of GLYMO and 4-hydroxy phenyl boronic acid (HPBA) on CuONPs we can produce formulations which are several times more effective against algae and yeast compared to bare CuONPs at the same conditions and particle concentration. The HPBA coating produces a surface functionality that allows the CuONPs particles to reversibly form covalent bonds with the *cis*-diol groups from glycoproteins and carbohydrates expressed on the cell wall of both yeast and algae. We show the profound differences in the surface properties of the bare CuONPs and the CuONPs/GLYMO/4-HPBA particles which have opposite surface charge at pH 5–6. The zeta potential of non-functionalized CuONPs, GLYMO-grafted CuONPs and HPBA-grafted CuONPs was +37 mV, −3 mV and −10 mV, respectively. Our tests showed that the anionic CuONPs/GLYMO/4-HPBA exhibit much higher anti-algal and anti-yeast action than the cationic bare CuONPs. This is explained by the strong covalent binding of the anionic particles CuONPs/GLYMO/4-HPBA to the cell walls due to formation of boronic ester bonds between 4-hydroxyphenylboronic acid and diol groups from carbohydrates expressed on the cell surface. SEM and TEM images of both sectioned *C. reinhardtii* and *S. cerevisiae* cells exposed to CuONPs/GLYMO/4-HPBA confirmed the significant accumulation of these nanoparticles on the cell membrane. Control experiments proved that the binding ability of the CuONPs/GLYMO/4-HPBA to algae and yeast can be adjusted and reversed by adding glucose in the media which competes for the HPBA groups of the CuONPs surface and reduces their ability to attach to the cell membrane. This effect allows direct control over their anti-algal and anti-yeast action. We also did experiments of incubation of the CuONPs/GLYMO/4-HPBA with human embryonic kidney cells which surprisingly showed very low cytotoxicity. We envisage that this type of surface coating can potentially be applied to a range of inorganic nanoparticles, as ZnONPs, TiO_2_NPs, Ag_2_ONPs, Cu_2_ONPs and others which would lead to fabrication of superior and more environmentally friendly anti-algal and antifungal agents at significantly reduced particle concentration.

## Conflicts of interest

The authors declare no conflict of interest.

## Abbreviations

CuONPs: Copper oxide nanoparticles
FDA: Fluorescein diacetate
GLYMO: (3-Glycidyloxypropyl)trimethoxysilane
4-HPBA: 4-Hydroxyphenylboronic acid.

## Supplementary Material

NA-001-C9NA00099B-s001
