# Supplementary material for: Self-grafting copper oxide nanoparticles show a strong enhancement of their anti-algal and anti-yeast action
Source: Nanoscale Adv. 2019 May 7;1(6):2323–36. doi: 10.1039/c9na00099b (PMC9417314; doi:10.1039/c9na00099b)
Supplement: NA-001-C9NA00099B-s001 [file NA-001-C9NA00099B-s001.pdf]

Electronic Supplementary Material (ESI) for Nanoscale Advances.
This journal is © The Royal Society of Chemistry 2019

Supplementary Information for:

Self-grafting copper oxide nanoparticles show a strong enhancement of their anti-algal and anti-yeast action

Ahmed F. Halbus,[1,2] Tommy S. Horozov,[1] Vesselin N. Paunov[1]*

[1] *Department of Chemistry and Biochemistry, University of Hull, Hull, UK;*
[2] *Department of Chemistry, College of Science, University of Babylon, Hilla, IRAQ.*

* Corresponding author contact details: Phone: +44(0)1482 465660, Fax: +44(0) 1482 466410; Email: v.n.paunov@hull.ac.uk

(Nanoscale Advances 2019)

Contents

The schematic of the synthesis method of CuONPs 1
The particle size and zeta potential of CuONPs 3
Fourier transform infrared spectroscopy (FTIR) analysis of CuONPs calcined at various temperatures 4
XRD pattern of CuONPs annealed at different temperature 6
Effect of the annealing temperature on the particles size and zeta potential of the CuONPs 7
EDX diagram of *C. reinhardtii* cells after treatment with CuONPs 9
EDX diagram of yeast cells treated with CuONPs 10
The zeta potential of *C. reinhardtii* suspensions treated with CuONPs 11
The zeta potential of *C. reinhardtii* suspensions treated with HPBA-grafted CuONPs...12
The zeta potential of *S. cerevisiae* suspensions treated with CuONPs 13
The zeta potential of *S. cerevisiae* suspensions treated with HPBA-grafted CuONPs...14
Anti-algal activity of GLYMO and 4-HPBA. 15
Anti- yeast activity of GLYMO and 4-HPBA. 16
Time-Kill assay statistical analysis on the data in Figure 4 17

The schematic of the synthesis method of CuONPs

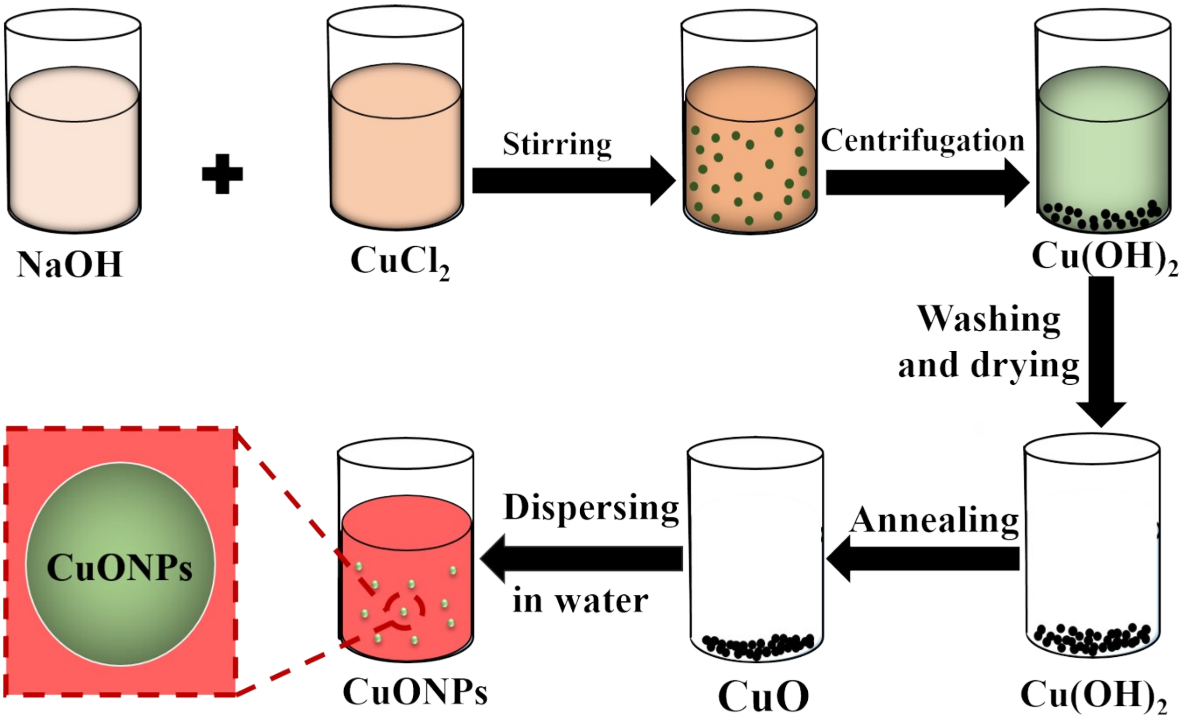

Figure S1. The schematic of the synthesis method of CuONPs.

The particle size and zeta potential of CuONPs

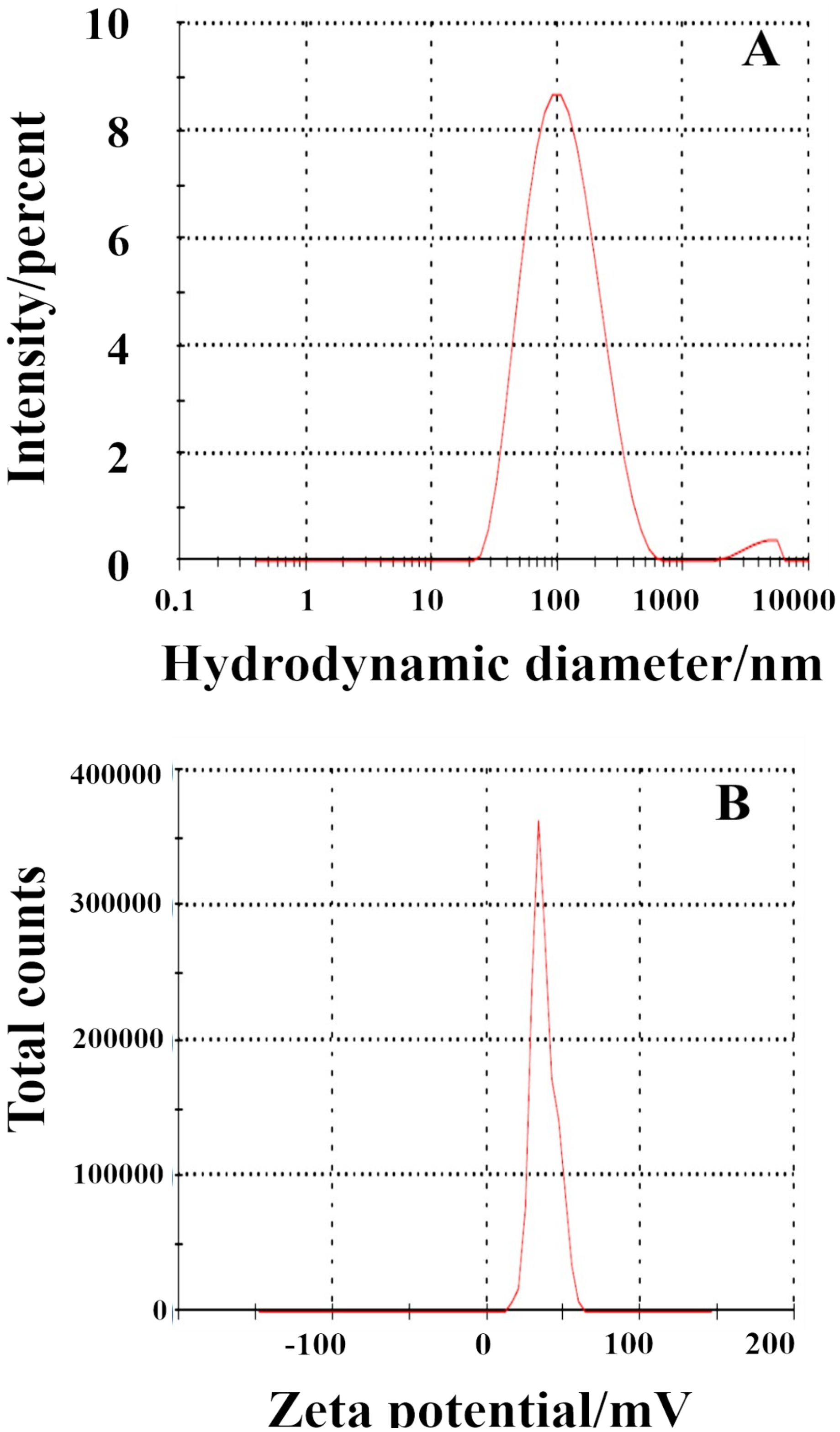

Figure S2. The (A) particle size and (B) zeta potential of CuONPs produced by annealing at 100 °C. The size and zeta potential of CuONPs was measured utilizing the Malvern Zetasizer NanoZL at room temperature with the average data of three readings.

Fourier transform infrared spectroscopy (FTIR) analysis of CuONPs calcined at various temperatures

Figure S3 presented the FTIR spectra of CuONPs annealed at 100^oC, 200^oC, 300^oC, 400^oC, 500^oC and 600^oC. The broad absorption peak at about 3445.89 cm^{-1} was caused by the adsorbed water molecules. Because of the nano crystalline materials possess a high surface to volume ratio, they can absorb moisture. Similar peak at 3434 cm^{-1} in the FTIR spectra of CuONPs are described.[34, 35] The peaks at 1633 might be for the Cu-O symmetrical stretching. [34, 36] The two infrared absorption peaks observed the vibrational modes of CuONPs in the range of 500 - 700 cm^{-1}. These peaks were detected at 533 cm^{-1} and 585 cm^{-1}, respectively. The peak at 533 cm^{-1} could be because of stretching of Cu-O.[37] The two peaks at 533 cm^{-1} and 585 cm^{-1} showed the creation of the CuONPs. These two peaks provision the existence of monoclinic phase. No other IR active modes are detected in the range of 500-700 cm^{-1}, which completely rules out the presence of Cu_2O. Two peaks at 525 cm^{-1} and 580 cm^{-1} in the FTIR spectra described for CuONPs which closely matches with our results.[38] Thus, the metal-oxygen frequencies observed for CuONPs are in near agreement with that of literature values.[34]

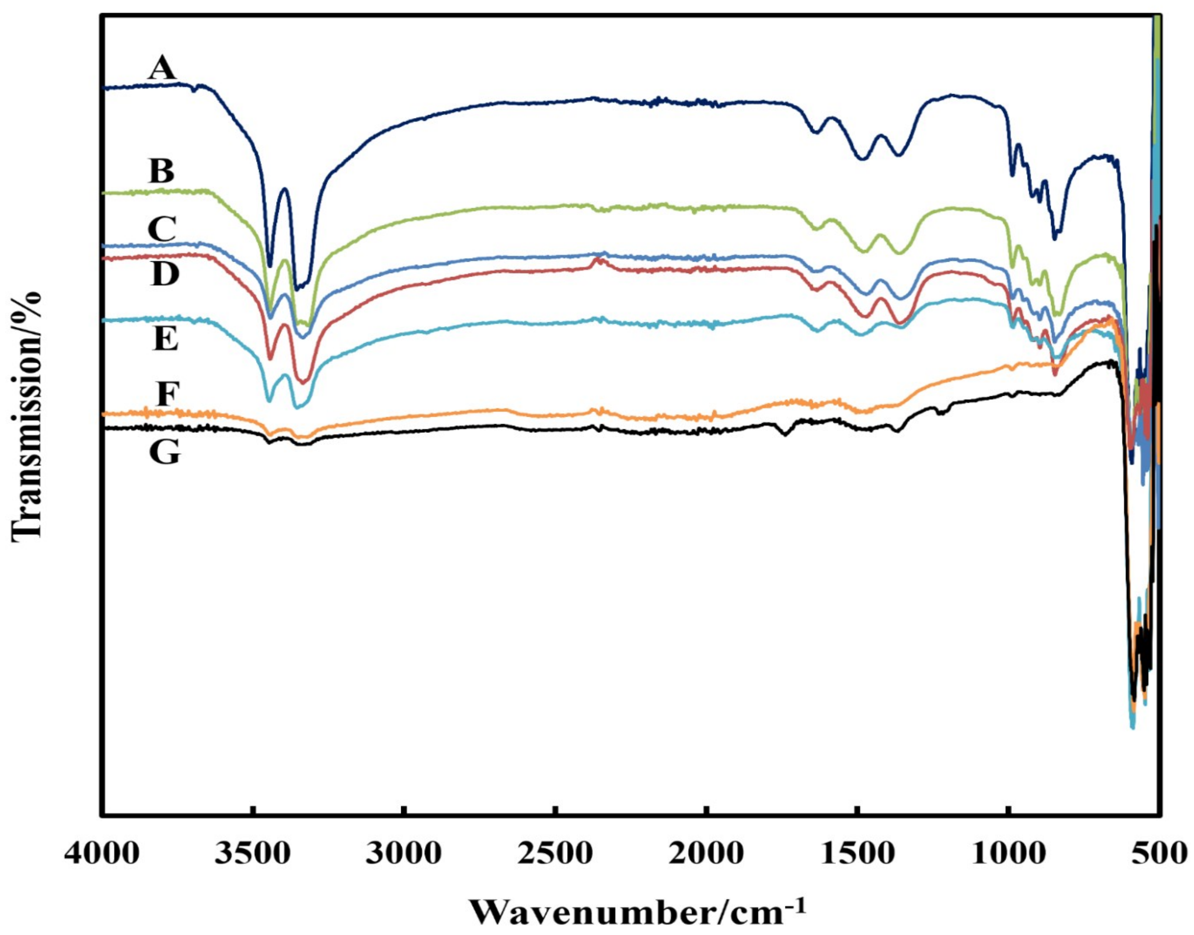

Figure S3. FTIR spectra of prepared CuONPs at different calcination temperatures (A) $Cu(OH)_2$ without calcinated , (B) 100 °C, (C) 200 °C, (D) 300 °C, (E) 400°C, (F) 500°C and (G) 600°C in the range of 500– 4000 cm^{-1}.

XRD pattern of CuONPs annealed at different temperature

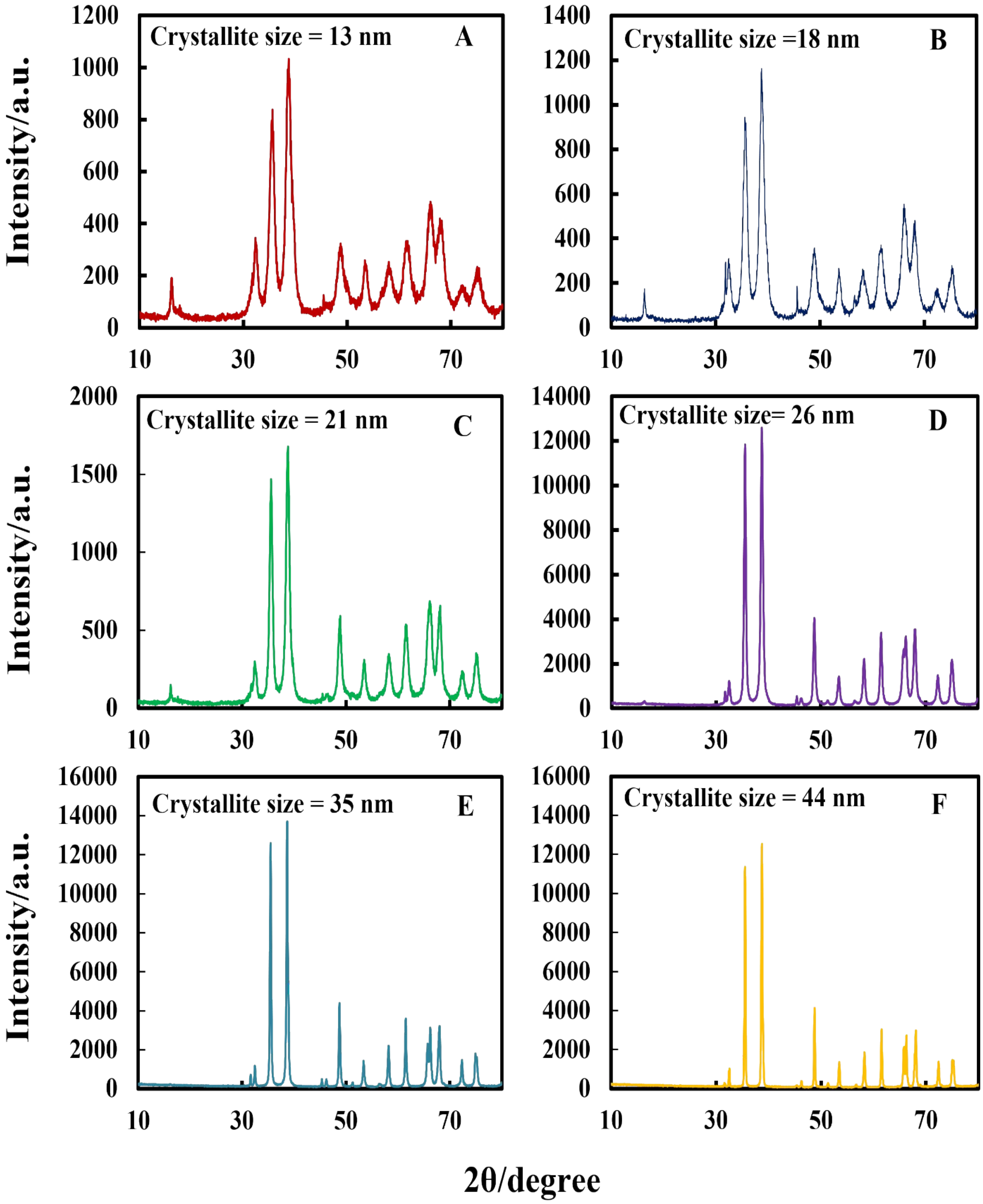

Figure S4. XRD pattern of CuONPs annealed at (A) 100 °C, (B) 200 °C, (C) 300 °C, (D) 400 °C, (E) 500 °C and (F) 600 °C with different crystallite size. The largest peak in the XRD results was applied to measure the crystallite size.

Effect of the annealing temperature on the particles size and zeta potential of the bare CuONPs

The particle size and zeta potential of CuONPs were examined at different calcination temperature as appeared in Figure S4 and Figure S5. From Figure S4, it is clear that the hydrodynamic diameter is increasing with increasing of the annealing temperature. Therefore, it was found that CuONPs with same crystal type but various particle size could be obtained by changing the calcination temperature and also these results were in agreement with the previous studies. These results may be explained that at higher calcination temperatures, the agglomeration of CuONPs begin to occur and hence the particle size increased. In addition to that, the zeta potential was tested for every calcined sample of CuONPs, and it can be seen from the Figure S5 that at 100°C, the zeta potential was +37 mV which means it was a highly stable while, at 600°C, the zeta potential was -4 mV.

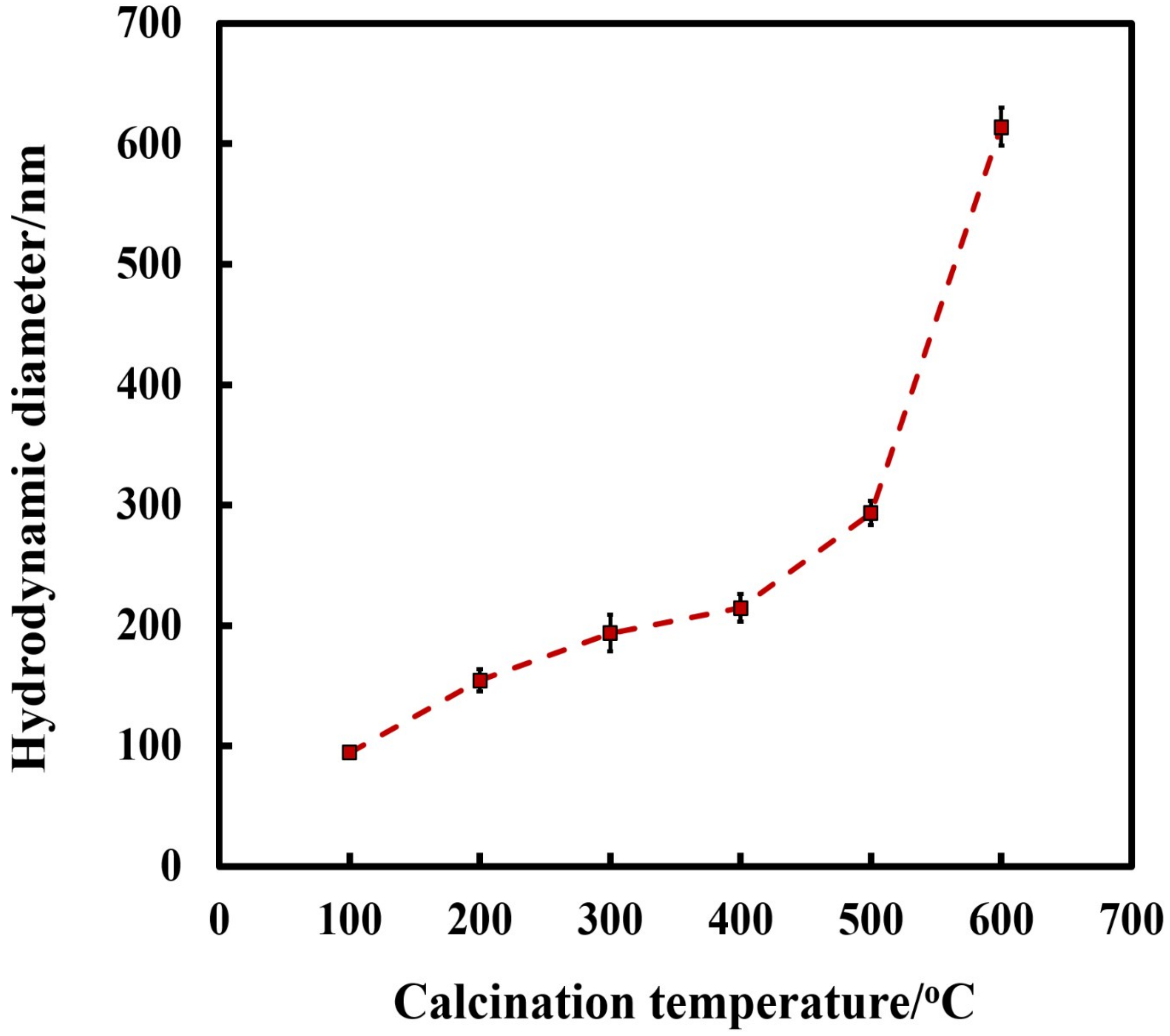

Figure S5. The hydrodynamic diameter of CuONPs annealed at various temperatures.

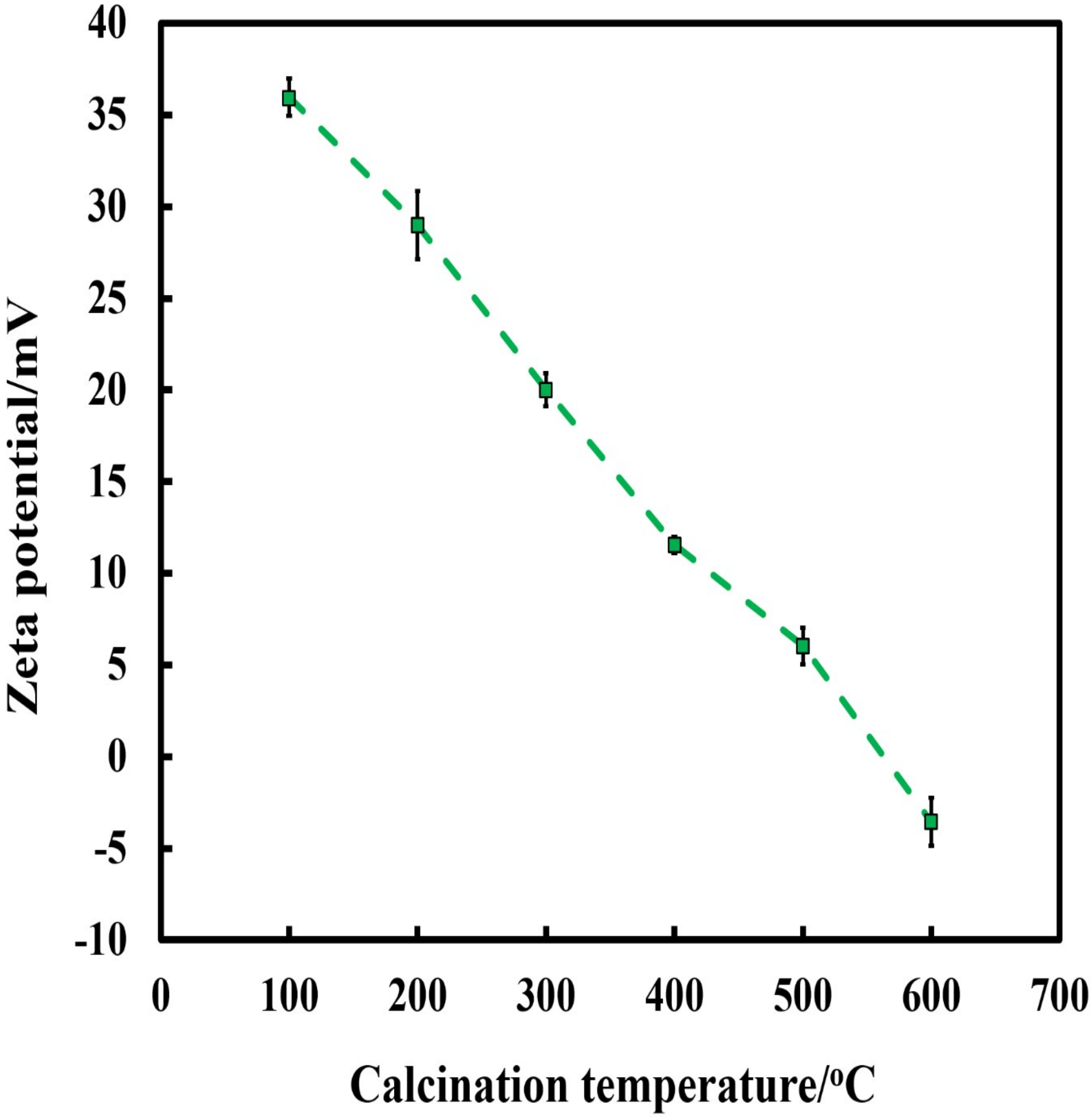

Figure S6. The zeta potential of bare CuONPs annealed at different temperatures.

EDX diagram of *C. reinhardtii* cells after treatment with CuONPs

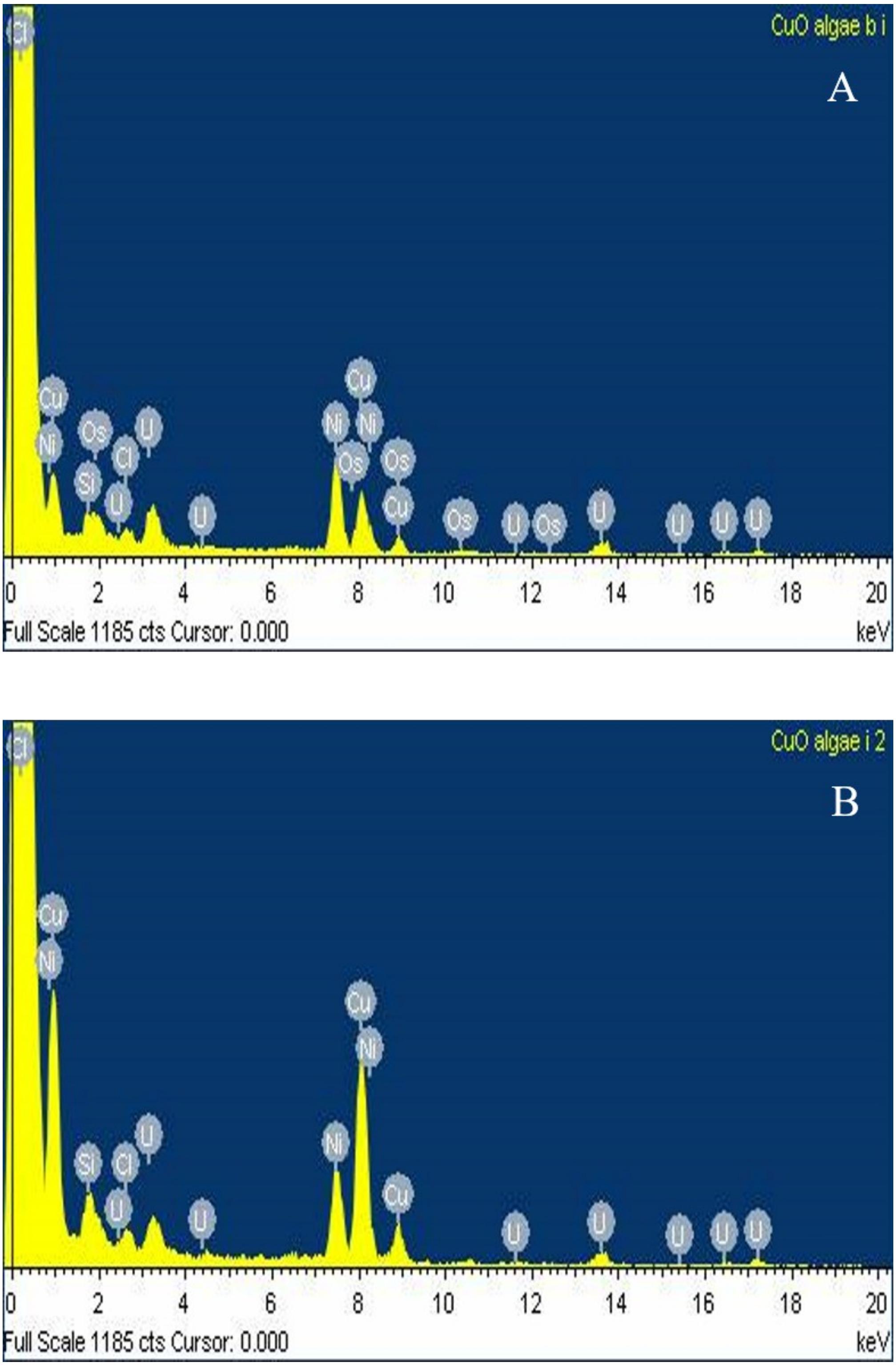

Figure S7. EDX diagram of *C. reinhardtii* cells treatment with CuONPs at 25 μg mL^{-1}: (A) *C. reinhardtii* inside membrane and (B) *C. reinhardtii* outside membrane areas. The result shows the existence of CuONPs on the inner and outer part of the cell membrane.

EDX diagram of yeast cells treated with CuONPs

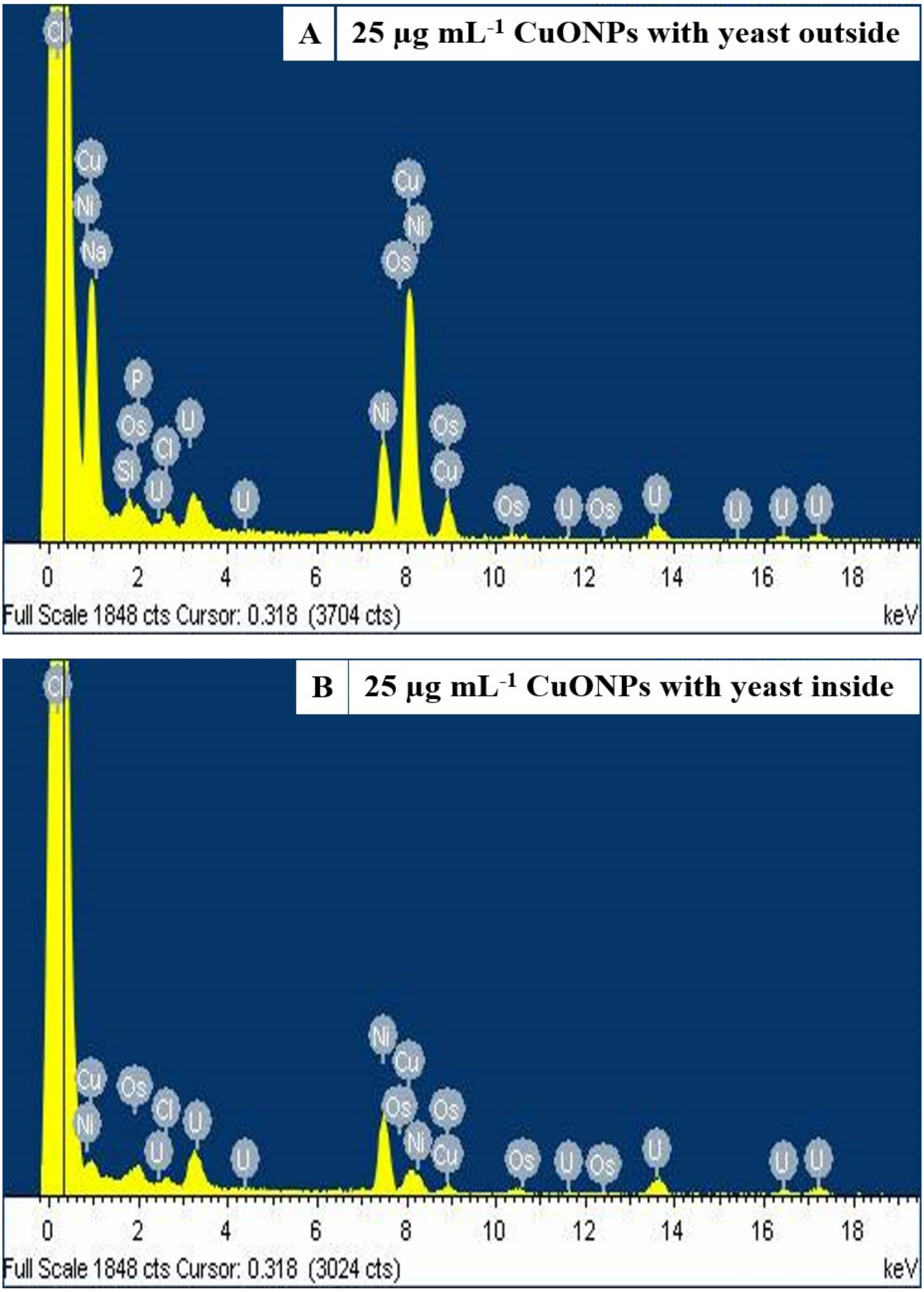

Figure S8. EDX diagram of *S. cerevisiae* cells with CuONPs at 25 µg mL^{-1}: (A) *S. cerevisiae* outside membrane and (B) *S. cerevisiae* inside membrane areas. The result shows the existence of CuONPs on the inner and outer part of the cell membrane.

The zeta potential of *C. reinhardtii* suspensions treated with CuONPs.

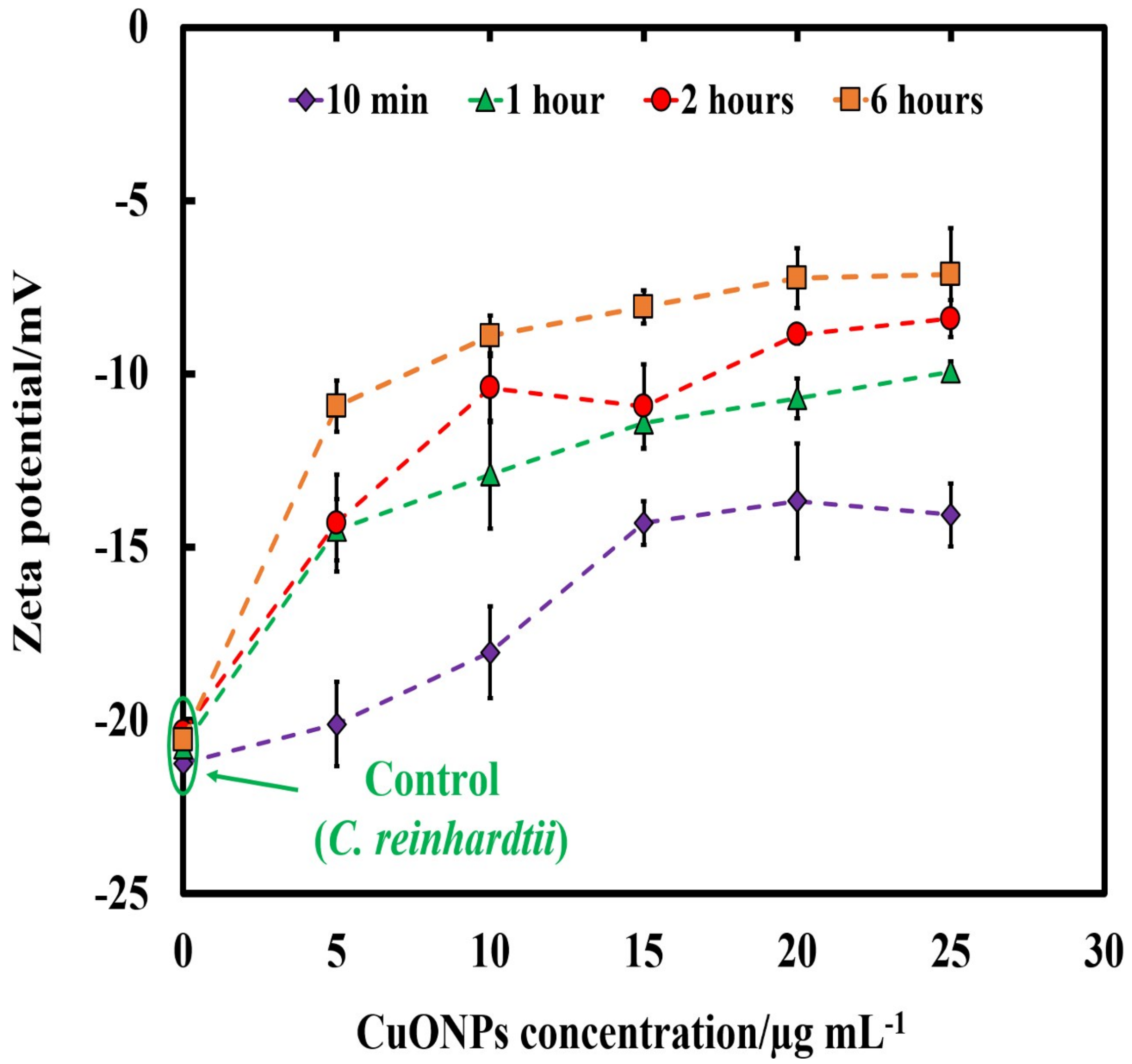

Figure S9. The zeta potential of *C. reinhardtii* suspensions treated with of various concentration of CuONPs at various exposure times. Error bars indicate standard deviations of means.

The zeta potential of *C. reinhardtii* suspensions treated with HPBA-grafted CuONPs.

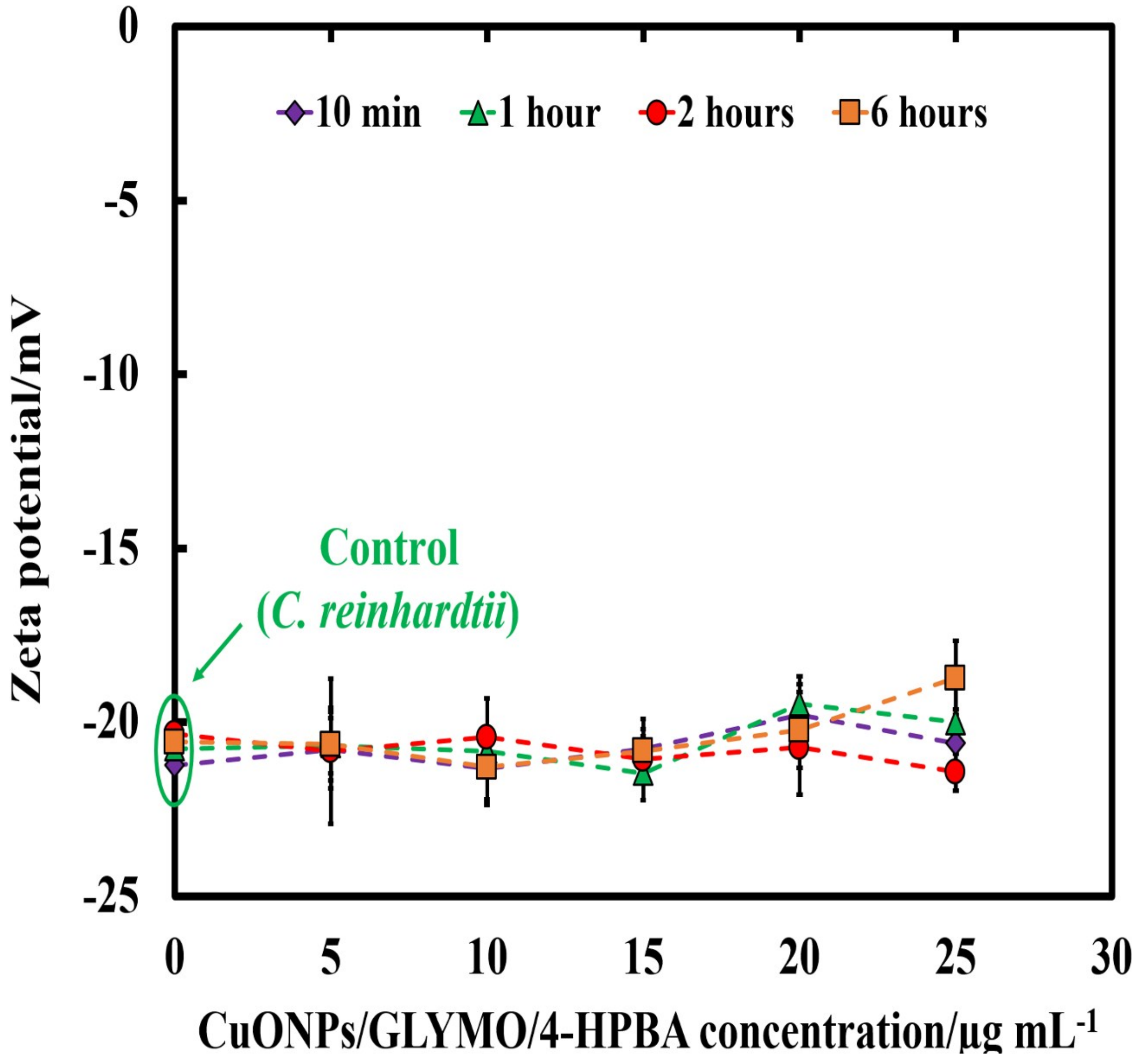

Figure S10. The zeta potential of *C. reinhardtii* suspensions treated with of various concentration of CuONPs/GLYMO/4-HPBA at various exposure times. Error bars indicate standard deviations of means.

The zeta potential of *S. cerevisiae* suspensions treated with CuONPs.

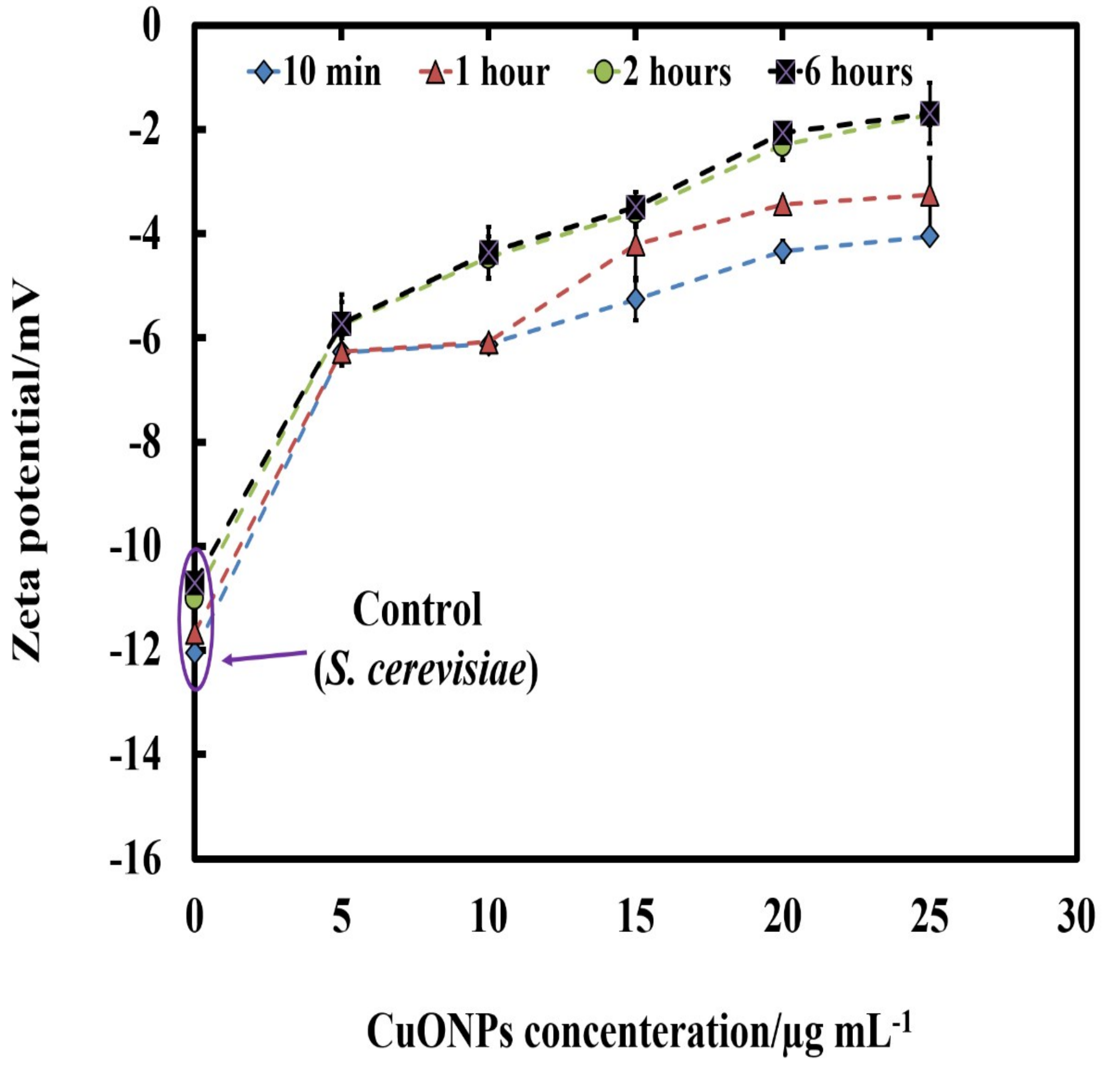

Figure S11. The zeta potential of *S. cerevisiae* suspensions treated with of various concentration of CuONPs at various exposure times. Error bars indicate standard deviations of means.

The zeta potential of *S. cerevisiae* suspensions treated with HPBA-grafted CuONPs.

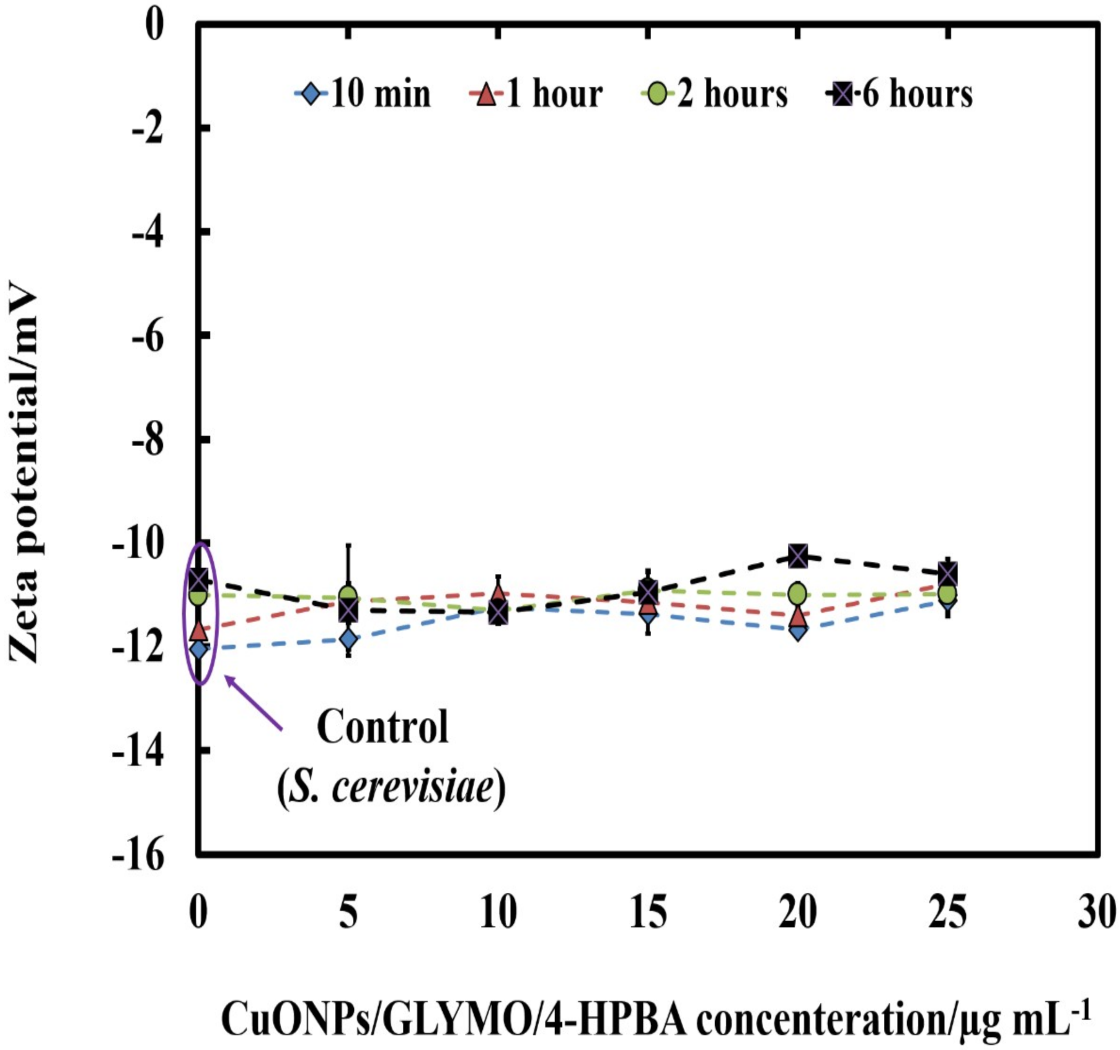

Figure S12. The zeta potential of *S. cerevisiae* suspensions treated with of various concentration of CuONPs/GLYMO/4-HPBA at various exposure times. Error bars indicate standard deviations of means.

Anti-algal activity of GLYMO and 4-HPBA.

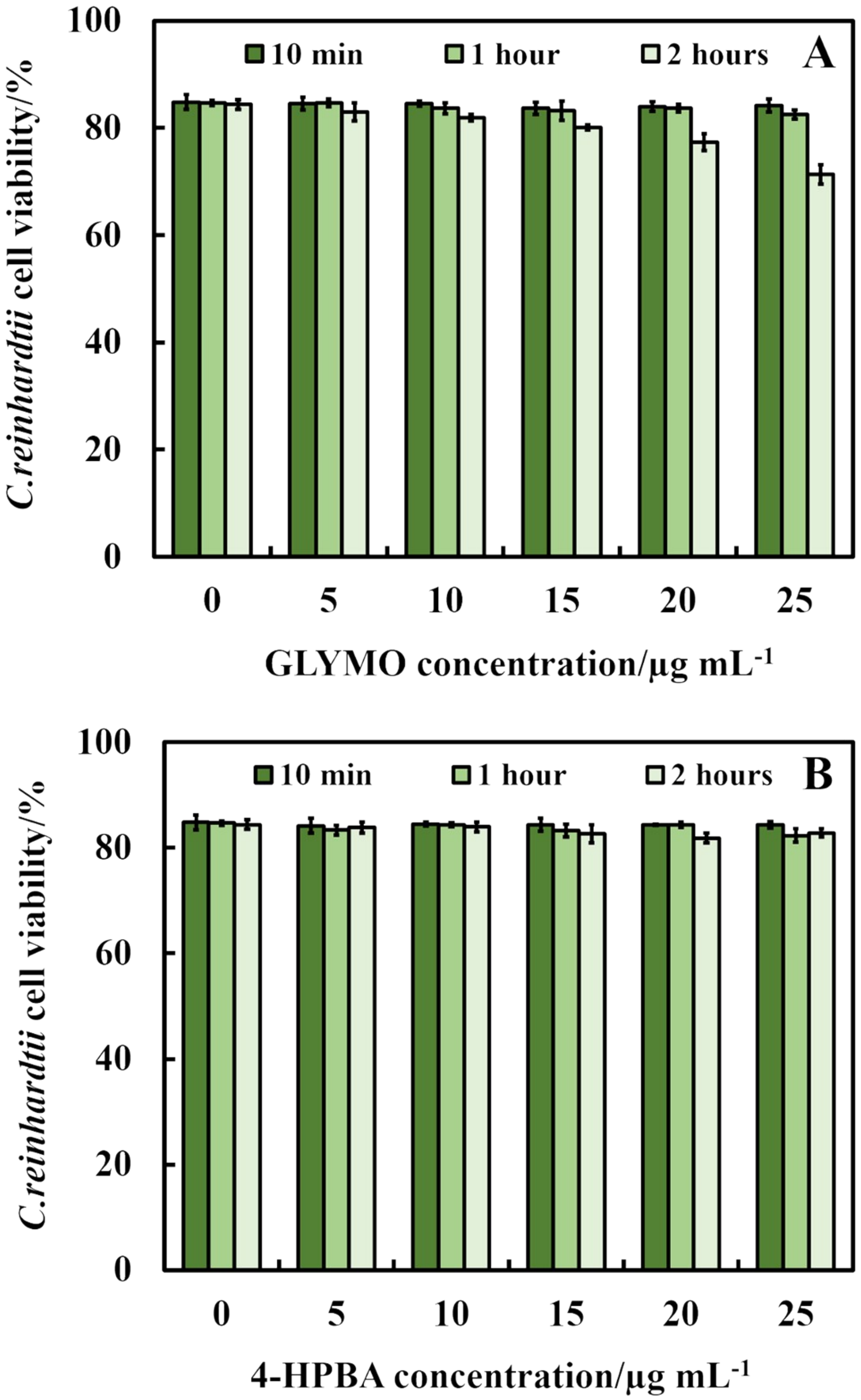

Figure S13. The anti-algal activity of (A) free GLYMO and (B) free 4-HPBA at various concentrations (0, 5, 10, 15, 20 and 25 μg mL^{-1}) on *C. reinhardtii*. The *C. reinhardtii* was incubated with the GLYMO and 4-HPBA at 10 min, 1 h and 2 h of exposure before being washed and tested for their cell viability.

Anti- yeast activity of GLYMO and 4-HPBA.

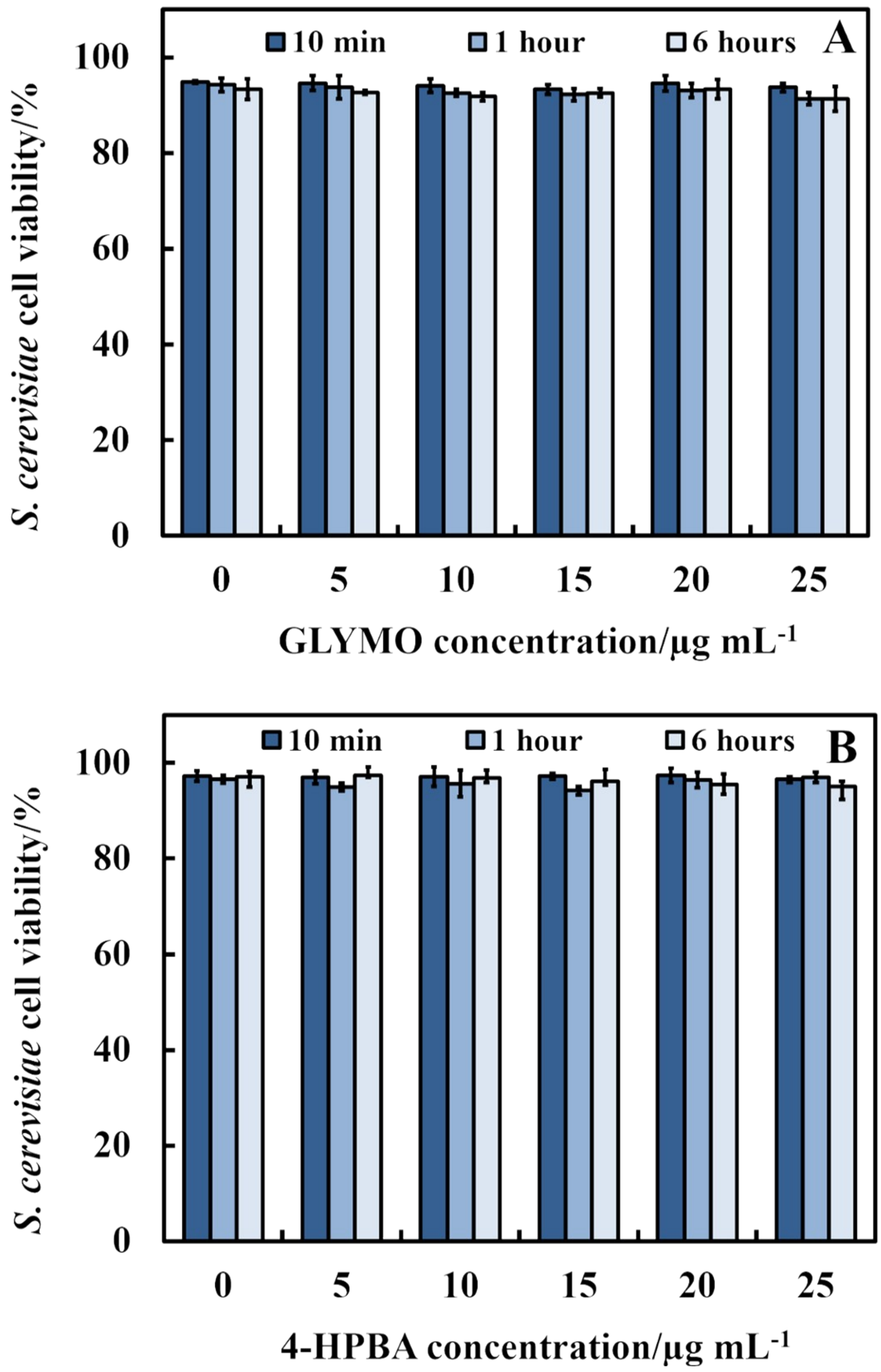

Figure S14. The anti-yeast activity of (A) free GLYMO and (B) free 4-HPBA at various concentrations (0, 5, 10, 15, 20 and 25 µg mL^{-1}) on *S. cerevisiae*. The *S. cerevisiae* was incubated with solutions of free GLYMO and free 4-HPBA at 10 min, 1 h and 6 h of exposure before being washed and tested for their cell viability.

Table S1. Time-Kill assay statistical analysis on the data in Figure 4 between bare, GLYMO or 4-HPBA-GLYMO-functionalized CuONPs at various concentrations (0, 5, 10, 15, 20 and 25 μg mL^{-1}) on *C. reinhardtii* at 10 min, 1 hour and 2 hours of exposure times in dark conditions, under visible and UV light. Data were expressed as average values ± standard deviations of the mean. P-values of less than 0.05 were considered significant.

**Species**	**Multiple Comparison**	**P-value**	**Significance**
C. reinhardtii	10 min bare CuONPs vs 10 min 4-HPBA-GLYMO-functionalized CuONPs in dark	0.000000039	***
	1 hour bare CuONPs vs 1 hour 4-HPBA-GLYMO-functionalized CuONPs in dark	0.000746407	***
	2 hours bare CuONPs vs 2 hours 4-HPBA-GLYMO-functionalized CuONPs in dark	0.022429609	*
	10 min bare CuONPs vs 10 min 4-HPBA-GLYMO-functionalized CuONPs under visible light	0.000068264	***
	1 hour bare CuONPs vs 1 hour 4-HPBA-GLYMO-functionalized CuONPs under visible light	0.030751950	*
	2 hours bare CuONPs vs 2 hours 4-HPBA-GLYMO-functionalized CuONPs under visible light	0.051276850	*
	10 min bare CuONPs vs 10 min 4-HPBA-GLYMO-functionalized CuONPs under UV light	0.000000020	***
	1 hour bare CuONPs vs 1 hour 4-HPBA-GLYMO-functionalized CuONPs under UV light	0.065300820	-
	2 hours bare CuONPs vs 2 hours 4-HPBA-GLYMO-functionalized CuONPs under UV light	0.133708000	-
	10 min GLYMO-functionalized CuONPs vs 10 min 4-HPBA-GLYMO-functionalized CuONPs in dark	0.000000001	***
	1 hour GLYMO-functionalized CuONPs vs 1 hour 4-HPBA-GLYMO-functionalized CuONPs in dark	0.000251755	***
	2 hours GLYMO-functionalized CuONPs vs 2 hours 4-HPBA-GLYMO-functionalized CuONPs in dark	0.001793596	**
	10 min GLYMO-functionalized CuONPs vs 10 min 4-HPBA-GLYMO-functionalized CuONPs under visible light	0.000025410	***
	1 hour GLYMO-functionalized CuONPs vs 1 hour 4-HPBA-GLYMO-functionalized CuONPs under visible light	0.000186613	***
	2 hours GLYMO-functionalized CuONPs vs 2 hours 4-HPBA-GLYMO-functionalized CuONPs under visible light	0.004923197	**
	10 min GLYMO-functionalized CuONPs vs 10 min 4-HPBA-GLYMO-functionalized CuONPs under UV light	0.000000005	***
	1 hour GLYMO-functionalized CuONPs vs 1 hour 4-HPBA-GLYMO-functionalized CuONPs under UV light	0.002375242	**
	2 hours GLYMO-functionalized CuONPs vs 2 hours 4-HPBA-GLYMO-functionalized CuONPs under UV light	0.003316280	**
	10 min bare CuONPs vs 10 min GLYMO-functionalized CuONPs in dark	0.940036000	-
	1 hour bare CuONPs vs 1 hour GLYMO-functionalized CuONPs in dark	0.078833000	-
	2 hours bare CuONPs vs 2 hours GLYMO-functionalized CuONPs in dark	0.116148000	-
	10 min bare CuONPs vs 10 min GLYMO-functionalized CuONPs under visible light	0.105017000	-
	1 hour bare CuONPs vs 1 hour GLYMO-functionalized CuONPs under visible light	0.060348000	-
	2 hours bare CuONPs vs 2 hours GLYMO-functionalized CuONPs under visible light	0.081441000	-
	10 min bare CuONPs vs 10 min GLYMO-functionalized CuONPs under UV light	0.298251000	-
	1 hour bare CuONPs vs 1 hour GLYMO-functionalized CuONPs under UV light	0.140810000	-
	2 hours bare CuONPs vs 2 hours GLYMO-functionalized CuONPs under UV light	0.092962000	-

< 0.05 is considered significant. *P <0.05, **P <0.01, ***P <0.001
